# Rising Global Riverine Deoxygenation Rates and GHG Emissions Driven by the Synergistic Effects of Warming and Anthropogenic Land Use Expansion

**DOI:** 10.1111/gcb.70828

**Published:** 2026-03-27

**Authors:** Ricky Mwangada Mwanake, Elizabeth Gachibu Wangari, Ralf Kiese

**Affiliations:** ^1^ Karlsruhe Institute of Technology, Institute for Meteorology and Climate Research Atmospheric Environmental Research (IMK‐IFU) Garmisch‐Partenkirchen Germany

## Abstract

Global fluvial ecosystems are increasingly impacted by human activities, such as climate warming and land use changes; however, the combined effects of these pressures on river greenhouse gas (GHG) supersaturation and deoxygenation remain poorly understood. This study modeled past global trends (2002–2022) in river GHG saturation, dissolved oxygen (DO) levels, water temperature, and eight other water quality parameters using machine learning models powered by satellite observations. Our findings show significant global increases in river GHG supersaturation and deoxygenation, mainly driven by rising water temperatures (0.27°C ± 0.03°C per decade), increased precipitation, higher labile carbon and nitrogen inputs, and urban and cropland expansion. We estimate that anthropogenic GHG emissions from rivers due to these pressures totaled 1.5 Pg‐CO_2_‐eq over the 20‐year period. The increase in GHGs was accompanied by a global river deoxygenation rate of 0.058 ± 0.01 mg L^−1^ per decade, suggesting rivers may be losing oxygen up to 2.5 times faster than lakes and oceans globally.

## Graphical abstract

Conceptual diagram summarizing study findings. Climatic shifts (river warming and intensified precipitation), together with expanding anthropogenic land uses, have driven elevated riverine GHG emissions and deoxygenation over recent decades. These responses reflect enhanced microbial activity under nutrient and organic matter enrichment at higher temperatures. Numbers indicate decadal estimates of warming, DO decline, and anthropogenic fractions of riverine GHG fluxes attributed to climatic and land‐use changes.

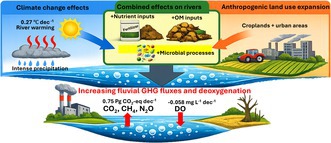

## 1 Introduction

Rising greenhouse gas (GHG) emissions and deoxygenation in streams and rivers have emerged as critical environmental concerns, driving global climate change and threatening freshwater habitats (Lauerwald et al. 2023; Zhi et al. 2023). Fluvial ecosystems are substantial contributors to global GHG budgets, emitting ~5 Pg (3.5–8.0) of CO_2_‐equivalent emissions annually (Lauerwald et al. 2023). The most significant contribution of these emissions comes from carbon dioxide (CO_2_), while methane (CH_4_), and nitrous oxide (N_2_O) emissions represent minor contributions (Lauerwald et al. 2023). In contrast to fluvial GHG concentrations, riverine oxygen concentrations are mostly undersaturated (Piatka et al. 2021), which can alter biogeochemical processes rates, exacerbate riverine GHG emissions, and lead to the proliferation of anaerobic conditions that are toxic to most freshwater organisms (e.g., Jane et al. 2021; Zhi et al. 2023).

Fluvial GHG emissions and deoxygenation are influenced by a variety of interconnected factors, spanning both natural and anthropogenic drivers. For instance, organic matter inputs to rivers from terrestrial ecosystems and wastewater discharge fuel microbial respiration and decomposition processes in rivers, leading to the production of CO_2_ and CH_4_ (Battin et al. 2023; Stanley et al. 2016). Additionally, nitrogen inputs from excessive fertilizers and urban wastewater can drive nitrification and denitrification processes, resulting in elevated riverine N_2_O emissions (Mwanake et al. 2019, 2024; Quick et al. 2019). All these GHG production processes are closely linked to river deoxygenation, as microbial activity results in the depletion of DO, particularly in low‐gradient nutrient‐rich ecosystems (e.g., Mwanake et al. 2022; Piatka et al. 2021; Upadhyay et al. 2023). Under hypoxic conditions, anaerobic metabolism is also favored, further enhancing the production of CH_4_ and N_2_O through methanogenesis and incomplete denitrification (Quick et al. 2019; Stanley et al. 2016).

Human‐driven climatic and land use changes may further alter current rates of GHG production and DO consumption processes, resulting in increased riverine GHG emissions or deoxygenation (Battin et al. 2023; Zhi et al. 2023). On one hand, rising global temperatures due to climate change lead to warmer rivers or more extreme precipitation events (e.g., Thackeray et al. 2022). These changes may increase the metabolic rates of riverine microbial communities, by enhancing inputs and processing of allochthonous nutrients and carbon, thereby accelerating instream oxygen consumption and GHG production (Battin et al. 2023; Yvon‐Durocher et al. 2014). Warmer rivers may also result in reduced oxygen solubility, further enhancing deoxygenation, as has been reported in regional studies (Zhi et al. 2023) and in other global aquatic ecosystems (Schmidtko et al. 2017; Zhang et al. 2025). On the other hand, land use changes such as urbanization, deforestation, and agricultural expansion can increase the inputs of labile organic carbon and nutrients into freshwater systems, creating hotspots for GHG emissions and deoxygenation (e.g., Dai et al. 2024; Ho et al. 2022; Mwanake et al. 2023; Singh et al. 2025; Upadhyay et al. 2023). Previous studies have also shown that anthropogenic land uses tend to warm more rapidly than natural landscapes (Liu, Zhan, et al. 2022; Marcotullio et al. 2021; Meng et al. 2023), suggesting that these two environmental pressures may co‐occur.

Despite the likelihood that the expansion of anthropogenic land uses will amplify the positive effects of global warming on riverine GHG supersaturation and deoxygenation, the potential synergies between these factors remain poorly quantified. For instance, the interaction between warming temperatures and increased precipitation‐driven substrate loading from anthropogenic land uses could elevate metabolic pathways such as respiration and nitrification that result in GHG supersaturation and deoxygenation, potentially leading to their annual increases. Furthermore, such temporal trends in GHG supersaturation and deoxygenation are expected to vary spatially due to regional differences in global warming and land use changes, making it more difficult to collectively predict future global trends. Addressing these knowledge gaps is crucial for developing effective region‐specific mitigation strategies and safeguarding the health of global riverine ecosystems in the face of ongoing human‐driven environmental changes. Yet, globally distributed multiannual datasets on riverine GHG supersaturation and deoxygenation are currently lacking, which limits our ability to assess long‐term trends in these phenomena under constantly changing environmental conditions (Battin et al. 2023; Dean and Battin 2024).

**FIGURE 1:**
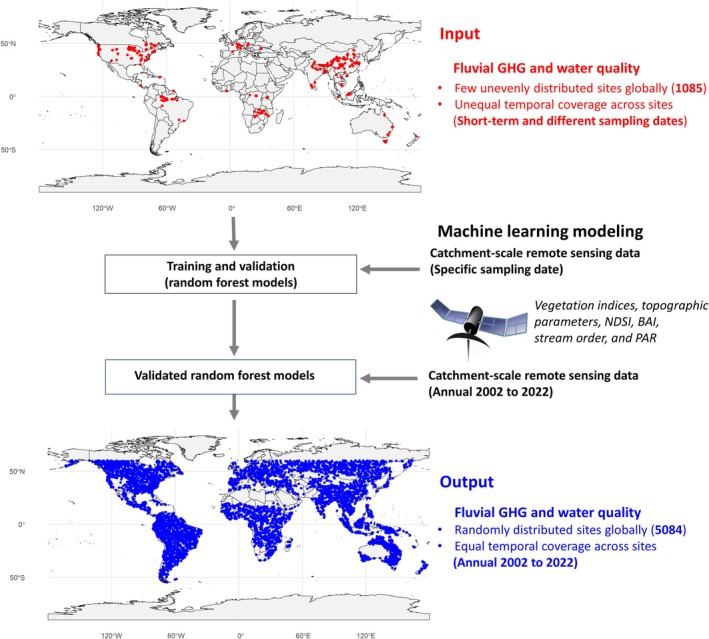
Flow diagram illustrating the workflow used to convert spatially and temporally uneven GHG and water‐quality observations into a spatially well‐distributed global dataset suitable for assessing long‐term trends in riverine GHG emissions and their underlying drivers. Map lines delineate study areas and do not necessarily depict accepted national boundaries. Empirical data from 1085 catchments (red) were combined with daily, remotely sensed predictors to train models that generated annual mean estimates for the period 2002–2022 across 5084 catchments (blue) randomly distributed worldwide.

To overcome these observational barriers, machine learning (ML) algorithms driven by remote sensing observations that describe catchment‐scale vegetation, climatic, and topographic characteristics have shown great promise in predicting the spatio‐temporal trends in riverine GHG concentrations and water quality with reasonable accuracy (e.g., Liu, Kuhn, et al. 2022; Mwanake, Wangari, et al. 2025; Rocher‐Ros et al. 2023; Xiong et al. 2024; M. Zhu et al. 2022). Mechanistically, such catchment‐scale characteristics reflect land nutrient, organic matter, and hydrological conditions (e.g., dos Santos et al. 2025; Farella et al. 2022; Imtiaz et al. 2024; Kunkel et al. 2022), which are known drivers of GHG and DO dynamics in streams, particularly during periods of land–stream hydrological connectivity (e.g., Liu, Kuhn, et al. 2022; Mwanake et al. 2022, 2023; Piatka et al. 2024). In this study, we adopted a comparable methodological framework and, for the first time, used daily satellite observations from the Moderate Resolution Imaging Spectroradiometer (MODIS) to train random‐forest machine‐learning models. This approach enabled modeling of global annual trends (2002–2022) in instream water temperature, GHGs, DO, and other critical water‐quality parameters across 5084 catchments randomly distributed worldwide (Figure 1). Our main objectives were (a) to determine the long‐term global trends in the modeled riverine GHG supersaturation and deoxygenation, (b) to determine the synergistic or antagonistic roles of warming rivers and the expansion of anthropogenic land uses in driving these long‐term global trends, and (c) to determine the relative contributions of climatic factors (water temperature and precipitation), anthropogenic land use (urban areas and croplands), and water quality parameters (dissolved oxygen, carbon, and nitrogen) to the long‐term temporal trends of fluvial GHG supersaturation at catchment scales.

## 2 Materials and Methods

### 2.1 Acquisition of the Training and Validation Data for the Machine Learning Modeling

Global input data for fluvial GHGs and water quality parameters were sourced from existing regional and global published datasets that reported both GHG concentrations and key water quality variables (Mwanake 2023b, 2023a; Stanley et al. 2023). The final dataset included water temperature, CO_2_, CH_4_, N_2_O, NH_4_, NO_3_, DOC, DO, TN, and TP concentrations from up to 1085 sites across six continental regions (South America, North America, Africa, Australia, Europe, Asia) sampled between November 2000 and April 2022 (Figures 1 and S1). These parameters were not consistently measured across all catchments, with TP being the least represented (Figures S1 and S2). Temporal sampling of GHGs and water quality parameters also varied: some sites were sampled only once, others multiple times within a year, and a few across multiple years (Figure S2). Overall, North America had the highest number of sites, Europe had, on average, the highest number of intra‐annually sampled sites for all parameters, while South America and Oceania lacked data for certain parameters (Figure S2).

All GHG concentration data were converted to percentage saturation using Equation (1) to account for global temperature variations and changes in atmospheric GHG concentrations during the sampling period. This calculation was done by normalizing their actual concentrations in the water (*C*_aq_ in moles L^−1^) with the stream water concentrations in equilibrium with varying atmospheric GHG concentrations (retrieved annual CO_2_, CH_4_, and N_2_O averages from 2000 to 2022 from https://gml.noaa.gov/ccgg/) (*C*_sat_ in moles L^−1^), calculated based on Henry's gas solubility constants at specific water temperatures and atmospheric pressure.

(1) $$\mathrm{GHG}\;\text{saturation}\;\left(\%\right)=\left(\frac{C_{\mathrm{aq}}}{C_{\mathrm{sat}}}\;\right)\times 100$$

For training the machine learning models, we relied exclusively on remote‐sensing (RS) predictors due to their wide spatial coverage (global extent) and high temporal resolution (daily), thereby overcoming the spatial and temporal limitations of land‐based observations (Figure 1). Although in situ water‐quality parameters can yield higher predictive performances for fluvial GHGs, previous work has shown that RS‐driven machine learning models achieve comparable mean absolute errors while offering the critical advantage of scalability (Mwanake, Wangari, et al. 2025).

To acquire the RS datasets for predicting water quality and GHG saturation trends, we first delineated the catchments for all 1085 sites (Figure 1). This delineation utilized the ArcGIS Pro (version 2024) watershed tool following several preprocessing steps. Firstly, we acquired the global flow direction raster file (15‐s resolution) and the global river network vector file from the HydroSHEDS database (https://www.hydrosheds.org/hydrosheds‐core‐downloads). We then used the snap tool in ArcGIS Pro to snap all 1085 sampling sites to the vertices of the global river network, setting a maximum snap distance of 50 m. Using the snapped points and the global flow direction raster as inputs, we delineated catchments for all global sites with the watershed tool in ArcGIS Pro. The resultant raster was subsequently converted to a polygon in ArcGIS Pro. Using these polygons, along with the specific sampling dates and site ID combinations from our combined global data as inputs, we automatically acquired several remote sensing (RS) datasets from Google Earth Engine (Figure 1; Table S1) with the R package “rgee” (version 1.1.7).

During the RS data acquisition, catchment‐scale means were computed for each sampling date and site, yielding daily numerical datasets for every stream reach (Figure 1). Because river surface areas, particularly those of small streams, are often too fine in resolution for directly matching them with remotely sensed data, reach‐scale means were instead calculated across the entire upstream catchment area. This approach has previously been successfully used to model riverine GHG trends using remotely sensed terrestrial products (Mwanake, Wangari, et al. 2025). The remotely sensed datasets used in the analysis were either temporally and spatially resolved (specific day and location) or only spatially resolved (Table S1). These included catchment‐scale means of daily vegetation indexes (Normalized Difference Vegetation index [NDVI], Normalized Difference Water Index [NDWI], Enhanced Vegetation Index [EVI]), the daily Normalized Difference Snow Index (NDSI), the daily Burn Area Index (BAI), the daily three‐hourly average photosynthetically active radiation (PAR) used individually for six timesteps, elevation, and a topographic diversity variable, which is a proxy for terrestrial biodiversity (Figure 1; Table S1). All daily indices derived from the MODIS database were corrected for atmospheric interference, and pixels affected by cloud cover were excluded (see MODIS User Guide v1.4: https://modis‐land.gsfc.nasa.gov/pdf/MOD09_UserGuide_v1.4.pdf).

Most of these RS parameters were selected for their potential to predict fluvial GHG and water quality dynamics, given their mechanistic linkages to nutrient and organic matter availability within the entire catchment, as well as hydrological exchanges between streams and the surrounding terrestrial landscape (e.g., Mwanake, Wangari, et al. 2025; Rocher‐Ros et al. 2023; Xiong et al. 2024). The selected parameters also mainly have daily and global coverage, which aligns with the sampling scales of the compiled global dataset on riverine parameters. In addition to this remotely sensed dataset, stream order (an indicator of stream size) was included as a potential predictor for GHGs and water quality parameters, as it controls terrestrial‐stream water connections (Butman and Raymond 2011; Hotchkiss et al. 2015; M. Li et al. 2021). The final dataset for model training and validation comprised measured GHG and water‐quality parameters, stream order, and remotely sensed catchment averages for each site on each sampling date (Figure 1; Table S1).

### 2.2 Construction of Global Random Forest Models to Predict Daily Fluvial GHG% Saturation and Water Quality From Remotely Sensed Data

For the prediction of daily riverine GHG % saturation (CO_2_, CH_4_, and N_2_O) and various water quality parameters (water temperature, conductivity, DO, pH, NH_4_^+^, NO_3_^−^, DOC, TN, SRP, and TP concentrations), we used the random forest algorithm built in the “CARET” (Classification And REgression Training, version 7.0–1) package in R (Figure 1). The rationale for using the random forest algorithm was that it has demonstrated comparable or better performance than other machine learning algorithms in predicting both GHG and water quality parameters (e.g., Liu, Kuhn, et al. 2022; Mwanake, Wangari, et al. 2025; Rocher‐Ros et al. 2023; Sakaa et al. 2022; J. Xu et al. 2021). To assess predictor importance at the catchment scale, we applied SHAP (Shapley additive explanations) using the “iml” package in R, which quantifies each predictor's contribution to model predictions. We then aggregated the SHAP values across all sites to obtain a global average ranking of predictor importance. From this global summary, we identified the three most influential predictors for each model and explored their relationships with predicted outcomes using partial dependency plots (“pdp” package in R).

During model construction, the global input dataset was randomly split into training (70%) and test (30%) sets, without regard to specific sites or sampling dates, enabling the generation of a generalized model across space and time. In addition to this hold‐out approach for model validation, we implemented a 10‐fold cross‐validation scheme on the training dataset using the “trainControl” function to internally validate our global models and mitigate overfitting (Berrar et al. 2019). By using the “set.seed” function, we specified a seed value of 142 to ensure the reproducibility of the model results across multiple runs. We automatically tuned the most critical hyperparameter (mtry), which represents the number of variables in each prediction tree node within the CARET package. This tuning process involved analyzing the sensitivity of the mtry value to changes in the mean absolute error (MAE) over 10 iterations and selecting the mtry value that yielded the lowest MAE. To improve model predictions for high values of GHG saturation and water quality data, we applied a natural log transformation to most parameters (except DO mg L^−1^, pH, and water temperature), which were not normally distributed and were skewed toward relatively low values.

In addition to the mean absolute error (MAE), model performance was evaluated by comparing observed and predicted values on the 30% test dataset excluded during model construction. Because our models were designed to capture temporal dynamics, the temporal skill (intra‐annual and interannual) of the models was quantified using the Kling–Gupta Efficiency (KGE) metric across all sites. The advantage of the KGE metric is that it integrates correlation, bias, and variability into a single score, providing a more balanced evaluation by explicitly including these different measures of error. A KGE value of 1 indicates perfect agreement, while values above 0.5 are generally considered acceptable. We also evaluated temporal performance using multiyear data from the test dataset, focusing on aggregated annual trends in GHG and DO saturation.

### 2.3 Construction of Past (2002 to 2022) Global Catchment‐Scale Annual Trends of GHG Saturation Percentages and Water Quality Parameters

We used the daily‐trained and validated random forest models for each parameter to reconstruct the annual global trends in GHG% saturation and water quality for the period 2002–2022 based on remote‐sensing observations (Figure 1, Table S1). The rationale for this modeling experiment was that the training dataset spanned a broad global domain, from the Southern to the Northern Hemisphere, and included rivers of varying sizes across contrasting landscape and climatic settings, thereby capturing substantial spatial heterogeneity relevant for upscaling (Figure 1). We acknowledge that a key limitation of the training dataset was its limited temporal coverage, both intra‐annual and inter‐annual (Figures S1 and S2). However, because the models were trained on remotely sensed observations matched to the exact sampling dates, they learned robust relationships between instream parameters and spatially and temporally explicit remotely sensed predictors, which were then used to fill temporal gaps in the observational record and generate past annual trends. While this approach does not fully substitute for continuous long‐term measurements, it provides a first‐order framework for temporally extending sparse observations and evaluating large‐scale patterns that would be impossible to investigate due to data limitations.

First, to ensure global representation in the annual computed estimates, while also staying within the limits of cloud computing on google earth engine, we randomly sampled 8000 catchment boundaries (1000 from each of the eight major continental regions: Africa, Asia, North America, Europe, Australia, Arctic, Siberia, South America) from the global hydrobasin dataset (Level 12; retrieved July 1, 2024: https://www.hydrosheds.org/products/hydrobasins). Based on the Dinerstein et al. (2017) classification, we then eliminated catchments within the desert ecoregions that may have few or no rivers, resulting in 5800 nested catchments with upstream sub‐basin areas ranging from 0.1 to 489 km^2^, representing stream orders 0 to 10 of stream widths < 1 to > 1000 m, similar to our training dataset.

Second, we obtained catchment‐scale annual means (from 2002 to 2022) of daily vegetation indexes (NDVI, NDWI, EVI), the daily snow cover index (NDSI), the daily burn area index (BAI), the daily three‐hourly average photosynthetically active radiation (PAR), elevation, and a topographic diversity variable from Google Earth Engine using the “rgee” package (Figure 1; Table S1). The annual mean CO_2_, CH_4_, N_2_O, and DO % saturation, as well as other water quality parameters at the pour point of each catchment, were then predicted using the random forest models constructed in the previous subsection and remotely sensed catchment input data (Figure 1). At this stage, some of the 5800 catchments located in the furthest parts of the upper northern hemisphere, Arctic and Antarctic regions, lacked all the RS data, resulting in a final prediction of the temporal trends (2002–2022) in GHG saturation and water quality for 5084 catchments, which were also used for further analysis (Figure 1).

### 2.4 Estimation of Modeled Global Fluvial GHG Fluxes for the 2002 to 2022 Period

In addition to modeling catchment‐scale GHG saturation values, we estimated global median fluvial GHG fluxes to compare them with past estimates. This was done using global median GHG supersaturation values modeled in this study and published datasets of global stream surface areas and gas transfer velocities (Lauerwald et al. 2023; Liu, Kuhn, et al. 2022). Global daily diffusive fluxes of CO_2_, CH_4_, and N_2_O were first calculated from our modeled median supersaturation values relative to atmospheric equilibrium concentrations based on data spanning 2002–2022 (atmospheric data derived from NOAA, https://gml.noaa.gov/ccgg/). Specifically, global equilibrium concentrations (*C*_sat_, mass m^−3^) were calculated using Henry's law constants at modeled global water temperatures and an atmospheric pressure of 1 atm. Global fluvial concentrations (*C*_aq_, mass m^−3^) were then estimated by multiplying the equilibrium concentrations by the median supersaturation values. Daily global fluxes per unit area (mass m^−2^ day^−1^) were computed using Fick's law of gas diffusion (Equation 2), where a global mean gas transfer velocity (*k*) of 9.4 (m day^−1^) (Liu, Kuhn, et al. 2022) was used.

(2) $$\text{Flux}=k\times \left(C_{\mathrm{aq}}-C_{\mathrm{sat}}\right)$$

These daily median fluxes were then upscaled to global annual fluxes by multiplying by the global ice‐free river surface area (616,000 km^2^) (Lauerwald et al. 2023) and 365 days. For uncertainty assessment, we applied a bootstrap (1000 runs) procedure to the median supersaturation values, propagating each replicate through the flux calculation to generate confidence intervals for the median fluxes. Final uncertainties of the global median flux estimates were then quantified using error propagation by combining the bootstrap confidence intervals with model uncertainties for each gas (7.2% for CO_2_, 9.1% for CH_4_, and 6.6% for N_2_O; Table S3), along with fixed uncertainties of 50% for gas transfer velocity and 28% for river surface area changes due to seasonality (e.g., Mwanake, Wangari, et al. 2025).

### 2.5 Analysis of the Effects of Combined Water Temperature and Land Use Changes on the Modeled GHG Saturations and Water Quality

To analyze the combined effects of warming rivers and land use changes on GHG saturation and water quality, we used the 20‐year reconstructed dataset from this study (2002–2022) and land use changes documented in the HILDA+ database from 2002 to 2019 (Winkler et al. 2021). Because the HILDA+ dataset only went up to 2019, analyses involving the combined effects of land use and warming were limited to this period. Land use change data for the 5084 sub‐catchments were obtained by calculating the percentage of the main land use classes in HILDA+ within each catchment. For this analysis, we focused on two main anthropogenic land uses (cropland and urban areas), which have been shown to have the most substantial effects on GHG concentrations and water quality (Mwanake et al. 2019, 2023; Upadhyay et al. 2023; W. Xu et al. 2024), while also examining forest cover changes, which represent the dynamics of more natural ecosystems.

After obtaining catchment‐scale anthropogenic land use data, we applied two complementary approaches to investigate how their interaction with river warming influences fluvial GHG supersaturation and deoxygenation at catchment scales. First, we employed the rate of change (slope) in water temperature and anthropogenic land use to classify the 5084 catchments into two categories for each variable: (a) low versus high water temperature increase, and (b) low versus high anthropogenic land use expansion. To achieve this, we fitted linear regressions of annual water temperature and anthropogenic land use (cropland + urban areas) against year, separately for each catchment. The slope coefficients from these regressions represent the average annual rate of change in each variable. We then calculated the global median slope values across all catchments. Catchments with slopes below the median were classified as having low increases, while those with slopes equal to or above the median were classified as having high increases. This approach allowed us to categorize each catchment into one of four distinct groups: (a) low water temperature increases with low anthropogenic land use increases, (b) high water temperature increases with low anthropogenic land use increases, (c) low water temperature increases with high anthropogenic land use increases, and (d) high water temperature increases with high anthropogenic land use increases. The final category represented the greatest potential impact, as it reflected catchments experiencing the combined pressures of intense warming and expanding human land use. Under these conditions, elevated temperatures, together with nutrient and labile carbon inputs from anthropogenic land uses, are expected to enhance GHG production and increase oxygen demand, thereby amplifying stress on riverine ecosystems.

Following this classification, we applied a second, complementary approach to quantify how GHGs and DO respond to warming and anthropogenic land use pressures, both individually and in combination, across all catchments. In this framework, water temperature and anthropogenic land use (urban + cropland) percentages were normalized to a 0–1 scale, and a combined warming and land use pressure index was calculated by averaging their normalized values. Annual rates of change for the 2002 to 2019 period were then estimated for GHGs, DO saturation, warming, land use expansion, and their combined effect at each site. We subsequently correlated the rates of GHGs and DO with warming, land use expansion, and the combined pressure index. This enabled us to evaluate whether fluvial GHG and DO responses exhibited stronger slopes under single versus combined pressures, thereby distinguishing synergistic effects (reinforcing responses) from antagonistic effects (counteracting responses). Together, these two approaches were meant to provide complementary perspectives: the slope‐based classification highlighted spatial contrasts and identified hotspot categories of catchments under rapid change, while the normalized environmental pressure index captured the continuous scaling of biogeochemical responses across the full gradient of combined pressures.

### 2.6 Statistical Analysis of the Modeled Dataset

Simple bivariate linear regressions were employed to analyze significant (*p*‐value) temporal changes in global and continental annual mean values of GHG% saturation and water quality parameters for the period 2002–2022. Linear mixed effects models (“lme4” package in R version 4.3.2), followed by a Tukey post hoc test of least‐square means, were then applied to determine significant differences between the four classes representing catchment‐scale differences in land use and water temperature change levels from 2002 to 2019. In the models, the fixed effects were the four classes, while the random effects included latitudinal differences within each catchment.

To further analyze the drivers of temporal changes in global GHG supersaturation and DO saturation, we applied elastic net regression models at the catchment scale. Elastic net regression models were chosen because they effectively address multicollinearity among predictors while simultaneously ranking their relative importance, thereby enabling direct comparisons of both indirect and direct drivers of fluvial GHG supersaturation. Before this analysis, annual precipitation data for each catchment and year were aggregated from the CHIRPS dataset (https://developers.google.com/earth‐engine/datasets/catalog/UCSB‐CHG_CHIRPS_DAILY#bands) and included in the global data to investigate the effects of precipitation on riverine GHG supersaturation. This precipitation data combined satellite observations with field measurements and covered a quasi‐global extent (50 N to 50 S) (Funk et al. 2015). In the elastic net regression models, the fixed effects encompassed both the indirect (land use, water temperature, and precipitation effects) and direct (nutrients, organic carbon, and DO) drivers of instream CO_2_, CH_4_, and N_2_O saturation, as referenced in previous studies (Battin et al. 2023; Piatka et al. 2021; Quick et al. 2019; Stanley et al. 2016). Specifically, the indirect drivers included water temperature, the percentage of cropland areas, the percentage of urban areas, and catchment‐scale precipitation, while the direct drivers included DIN, dissolved organic nitrogen (DON) (calculated as the difference between TN and DIN), and DOC concentrations. Model fitting was performed using the “lamda.min” penalty parameter, which corresponds to the regularization parameter value that minimizes cross‐validated error. Site‐level coefficients were then averaged across all catchments, and their standard errors were calculated to provide robust estimates of effect sizes. Forest plots were then used to visualize these averaged standardized coefficients (“sjPlot” and “ggplot2” packages in R version 4.3.2).

## 3 Results and Discussion

### 3.1 Performance of the Global Random Forest Model Predictions Relative to Field Observations

Random forest models driven by both temporally and spatially resolved remotely sensed datasets were able to predict the spatio‐temporal trends in global riverine GHG saturation, DO concentration, and other water quality parameters (*p* < 0.05), with model performance ranging from moderate to high (*R* ^2^ = 0.50 to 0.90; Table S2). These results are consistent with findings from previous studies, which highlighted the effectiveness of machine learning models for the spatio‐temporal prediction of riverine parameters (Liu, Kuhn, et al. 2022; Marzadri et al. 2021; Mwanake, Wangari, et al. 2025; Rocher‐Ros et al. 2023; Zhi et al. 2023). When evaluating predictor importance, their relative influence varied across the response variables (Figure S3). Nevertheless, the top three predictors mainly yielded trends that matched their expected mechanistic links with GHGs, DO, and other water quality parameters (Figure S3).

**FIGURE 2:**
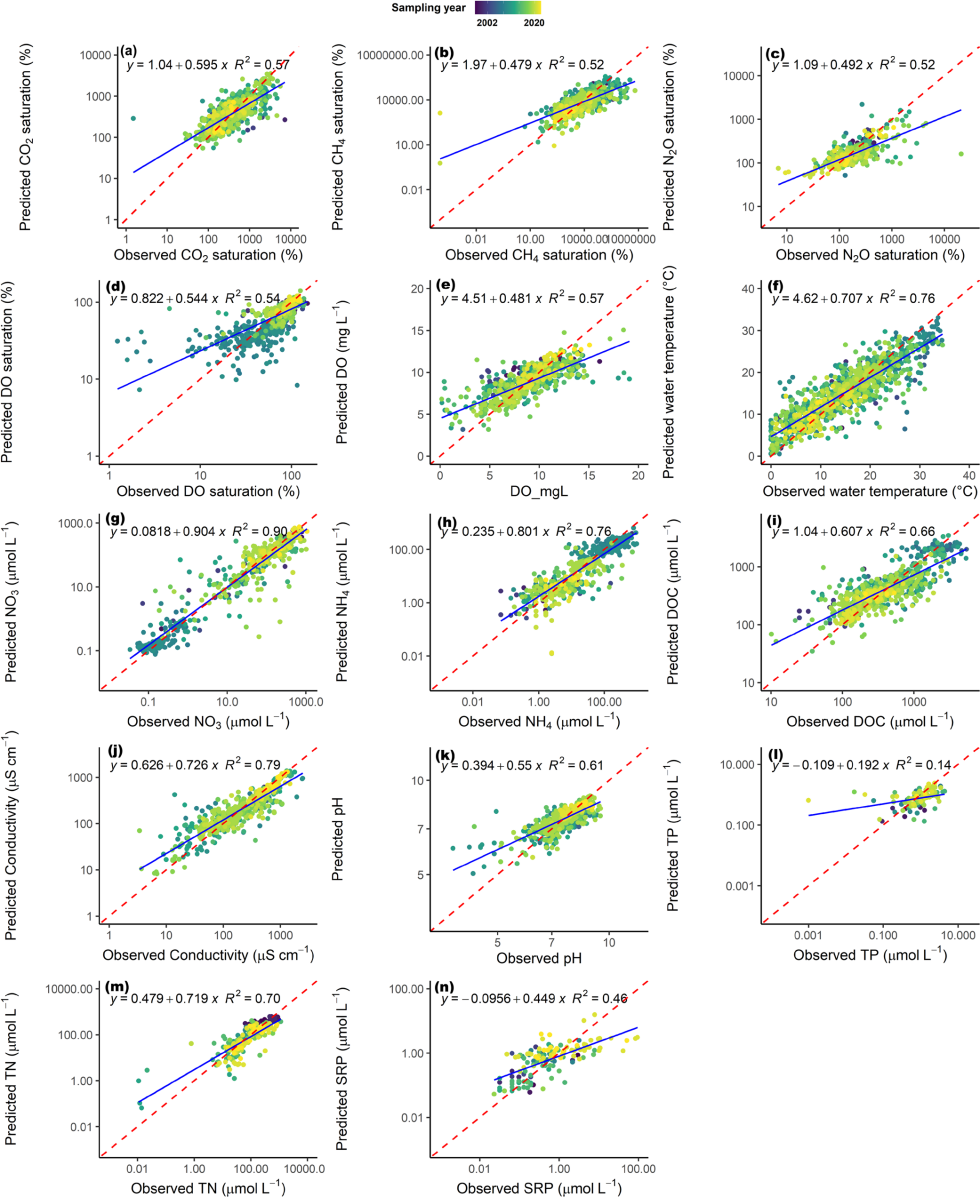
Regression analysis of the observed versus the predicted values of GHG saturation, DO saturation, and several water quality parameters from 30% of the data not used in model construction. The colors of the plots show predictions of day and site‐specific measurements from different sampling years, the dotted red line shows the 1:1 prediction, and the solid blue line shows the regression line.

The global annual means and ranges of the daily predicted values for GHGs, DO, and water quality parameters also fell within the range of observed measurements (Table S2), suggesting that the model outputs effectively captured the general spatio‐temporal patterns in the global dataset. Model uncertainties, expressed as the percentage of the mean absolute error (MAE) relative to the observed global means, ranged from 6.2% to 47.2% (Table S2). Overall, the lowest uncertainty was associated with DO predictions, while the highest uncertainty was observed for total phosphorus (TP), which had the fewest field measurements available for model training (Table S2). That said, the uncertainty values for GHG % saturation predictions were relatively low compared to the other predicted parameters, ranging from 6.6% for N_2_O to 7.2% for CO_2_ and 9.1% for CH_4_. The RF models trained on RS data also captured site‐scale parameter changes for specific sampling dates in the test dataset, as indicated by the comparison of observed versus predicted values (Figure 2). Based on this analysis, the RF models generally overestimated low values and underestimated high values, with the magnitude of these uncertainties varying by predicted parameter (Figure 2a–n). A further analysis of the general temporal skill (intra‐annual and inter‐annual) of the models at the site scale indicated mainly acceptable performance across all parameters (Figure S4; median KGE value = 0.54). When assessing interannual variability, the models were also mainly able to reproduce annual trends in water temperature, DO, and GHGs within the bounds of the MAE at the few sites with multi‐year data outside the training dataset (Figure S5).

### 3.2 General Spatial–Temporal (2002–2022) Patterns of Modeled Riverine CO_2_ , CH_4_ , N_2_O, and DO Concentrations

To assess long‐term temporal trends in riverine GHG concentrations and water quality parameters, we used our modeled dataset, which overcomes the limitations of measured observations, including uneven global site distribution and short, inconsistent temporal coverage (Figure 1). Throughout the two decades, modeled global mean annual saturations of CO_2_, CH_4_, and N_2_O were consistently supersaturated relative to atmospheric equilibrium concentrations (median: 400%, 6324%, and 146%, respectively), indicating that global fluvial ecosystems are net sources of these greenhouse gases (GHGs) to the atmosphere (e.g., Lauerwald et al. 2023). The spatial and temporal patterns in the GHG saturations also showed considerable variability, spanning up to four orders of magnitude, with CH_4_ exhibiting the greatest variability and N_2_O the least (Table S3; Figure S6). Regarding continental differences, the tropical regions of South America and Africa were hotspots for CO_2_ and CH_4_ supersaturation, respectively, while the supersaturation of N_2_O was highest in North America and South America (Figure S6), reflecting continental trends similar to field‐based observations (Lauerwald et al. 2023).

The estimated diffusive global fluvial GHG emissions from our modeled median supersaturation values for the entire 20‐year period were 1.32 Pg CO_2_‐C year^−1^ (0.56–2.09 Pg), 4.60 Tg CH_4_‐C year^−1^ (1.93–7.27 Tg), and 287 Gg N_2_O‐N year^−1^ (121–453 Gg). These median values are consistent with published global estimates for CO_2_ (0.5–3.2 Pg CO_2_‐C year^−1^), lower than reported estimates for CH_4_ (16.5–18.5 Tg CH_4_‐C year^−1^), and comparable to but slightly higher than estimates for N_2_O (45.18–283.18 Gg N_2_O‐N year^−1^) (Lauerwald et al. 2023). In terms of carbon dioxide equivalent emissions based on global warming potentials over 100 years, we estimated riverine median emissions of 5.15 Pg‐CO_2_‐eq year^−1^ for the entire period, which is also within the range of the annual CO_2_‐eq median fluxes (3.5–8.0) compiled by Lauerwald et al. (2023).

In addition to acting as GHG sources, global rivers also exhibited both supersaturation and undersaturation in DO, with a mean annual saturation over the two decades of 77% indicating a general undersaturation in most rivers (Table S3). This finding suggests that most rivers were predominantly net heterotrophic, consistent with global field observations indicating similar DO undersaturation in 84% of rivers, though with a higher average saturation of 93%, based on samples collected primarily during the day (Piatka et al. 2021). Spatially, the highest average fluvial DO saturation values were found in the cold Arctic and Siberia regions, while the lowest average DO saturation values were observed in the Australia region (Figure S6).

**FIGURE 3:**
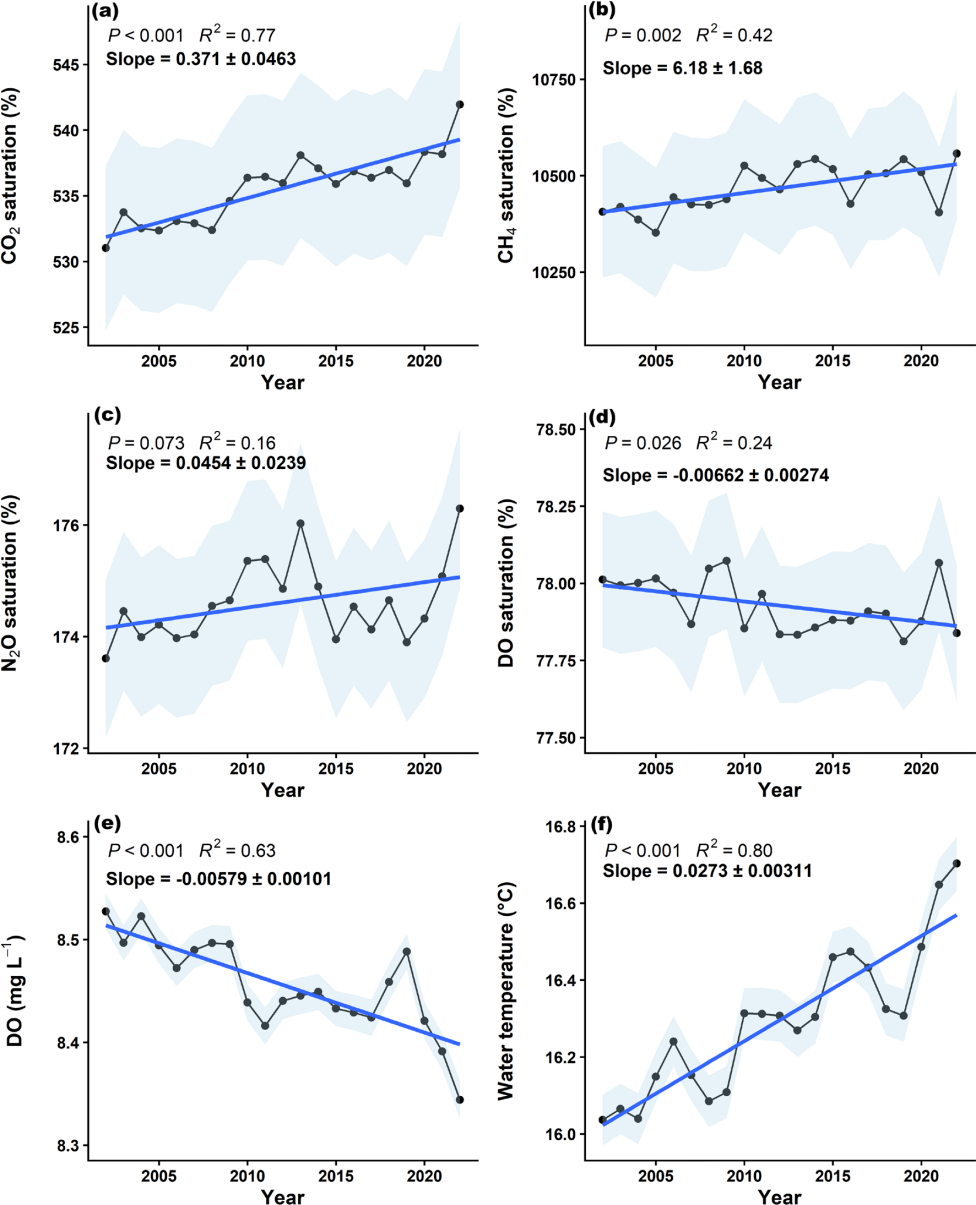
Global annual trends of mean (black line and dots) ± SE (grey shaded area) of CO_2_, CH_4_, N_2_O, and DO in the 5084 river reaches. The blue line shows the regression fit, with the slope ± SE of the linear fit in black. The GHG and DO trends represent modeled values from this study (2002 to 2022).

Beyond these general observations, our study also reveals notable increases in global riverine water temperature, GHG supersaturation (CO_2_, CH_4_, and N_2_O), and riverine deoxygenation over the past two decades (Figure 3a–f). These findings are consistent with previous modeling studies that have identified similar regional trends for CO_2_ and DO (Mu et al. 2025; Rajesh and Rehana 2022; Zhi et al. 2023), as well as global patterns for CO_2_ and N_2_O (Tian et al. 2023; Yao et al. 2020). The deoxygenation trends in global rivers are also consistent with patterns observed in global lakes and oceans, linked to both climatic and anthropogenic land use drivers (Schmidtko et al. 2017; Zhang et al. 2025). However, the global riverine deoxygenation rate estimated in this study (Figure 3e; 0.058 ± 0.01 mg L^−1^ dec^−1^) exceeds those reported for oceans (0.022 mg L^−1^ dec^−1^; Schmidtko et al. 2017) and lakes (0.049 mg L^−1^ dec^−1^; Zhang et al. 2025), indicating that rivers may be losing oxygen up to 2.5 times faster than these other aquatic systems. Mechanistically, rivers, particularly small streams, are likely more vulnerable to extreme weather events such as droughts and heavy precipitation pulses than lakes and oceans. These disturbances can elevate oxygen demand through heightened organic matter inflows following storms, or by increasing respiration rates under warmer, slow‐flow conditions. Graham et al. (2025), using a hybrid modeling framework, reported similar declines in global riverine DO from 1980 to 2019, although their estimated rate of 0.042 mg L^−1^ dec^−1^ was lower than what we report in this study. Differences between our studies may be due to their longer modeled period, which likely encompassed earlier decades of slower DO change, as well as methodological contrasts given their hybrid modeling approach.

Unlike previous studies that examined regional long‐term temporal trends in GHGs (Mu et al. 2025) or deoxygenation (Zhi et al. 2023) separately, the modeling of simultaneous changes in riverine GHG supersaturation and deoxygenation alongside other water quality parameters in this study allowed for a comprehensive assessment of shared and distinct drivers behind these temporal trends at larger global scales. Notably, we observed that rising global levels of GHG supersaturation and deoxygenation coincided with increases in global upstream anthropogenic land use (urban and cropland), stream water temperature, stream acidification, and stream conductivity, as well as declines in the dissolved organic carbon to total dissolved nitrogen (DOC:TN) ratio (Figures 3, S7, and S8). These concurrent patterns suggest positive effects of anthropogenic pressures on riverine GHG supersaturation or deoxygenation, driven by C:N stoichiometric changes due to anthropogenic N addition and climate warming, conclusions that align with prior research (Mu et al. 2025; Piatka et al. 2021; Rajesh and Rehana 2022; Tian et al. 2023; Yao et al. 2020; Zhi et al. 2023).

At the continental scale, the most pronounced temporal changes were observed in stream reaches within Asia, which includes rapidly developing countries such as China, India, Bangladesh, Pakistan, and Indonesia (Table 1). This continent experienced, on average, the highest annual increases in CO_2_ and N_2_O saturations and decreases in DO concentrations up to four times greater than those recorded in other regions (Table 1). These patterns also coincided with some of the highest rates of urbanization and river warming during the study period (Table 1). Based on these findings, we contend that intensified urbanization in the region may have increased nutrient and labile carbon loads in streams, which, together with rising water temperatures, likely enhanced CO_2_ and N_2_O production processes such as respiration, nitrification, and denitrification, while simultaneously accelerating oxygen consumption. This interpretation is in line with previous studies within the region that have directly linked urbanization or warming to elevated GHG levels and deoxygenation in fluvial systems (Dai et al. 2024; Rajesh and Rehana 2022; Ran et al. 2021; Singh et al. 2025; Wang et al. 2023).

**TABLE 1:** Mean continental decadal changes in land use from 2002 to 2019 and greenhouse gas, water temperature, and DO from 2002 to 2022. Only significant (*p* < 0.05 and *p* < 0.1*) decadal rates are shown.

Continent	Landuse			Greenhouse gases			Water quality	
	Urban (% dec^−1^)	Cropland (% dec^−1^)	Forest (% dec^−1^)	CO_2_ saturation (% dec^−1^)	CH_4_ saturation (% dec^−1^)	N_2_O saturation (% dec^−1^)	Water temperature (°C dec^−1^)	DO (mg L^−1^ dec^−1^)
Africa	0.053	0.71	−1.25	1.93	110.86			−0.022
Arctic of North America		0.19	−0.50	1.74*				
Asia	0.120	−0.15	0.32	7.00	−58.89	2.63	0.566	−0.083
Australia	0.299	1.54	−0.37	6.69	77.96		0.288	−0.077
Europe	0.179	−0.82	0.70	3.82			0.142	−0.058
North America	0.142	−0.76	0.48				0.164*	−0.040
Siberia		−0.20	−0.42	2.47			0.386	−0.030
South America	0.019	0.33	−1.30	2.98			0.399	−0.085

The Australian continent also exhibited some of the highest increases in urbanization, cropland expansion, river warming, and riverine CO_2_ and CH_4_ saturation, along with the steepest declines in riverine DO concentrations (Table 1). These trends agree with earlier findings that highlight substantial GHG emissions or deoxygenation in rivers in this region, attributed partly to elevated nutrients and bioavailable carbon inputs from agricultural activities (Guo et al. 2025; Woodrow et al. 2024). Other regions with significant annual trends in the modeled catchments included Europe (notably for CO_2_ supersaturation, deoxygenation, warming and urbanization), South America (CO_2_ supersaturation, warming, cropland and urban expansion), Africa (urban and cropland growth, forest cover declines, increases in CO_2_ and CH_4_ saturation and deoxygenation) and Siberia (CO_2_ supersaturation, forest cover declines, river warming and deoxygenation) (Table 1). In contrast, the North American region, which exhibited significant cropland decreases and forest cover increases, showed no notable increases in riverine GHG supersaturation. However, riverine deoxygenation was still observed in North America, likely linked to the marginally significant rates of river warming detected in these regions, but was absent in the Arctic region of North America, which lacked significant river warming rates (Table 1).

**FIGURE 4:**
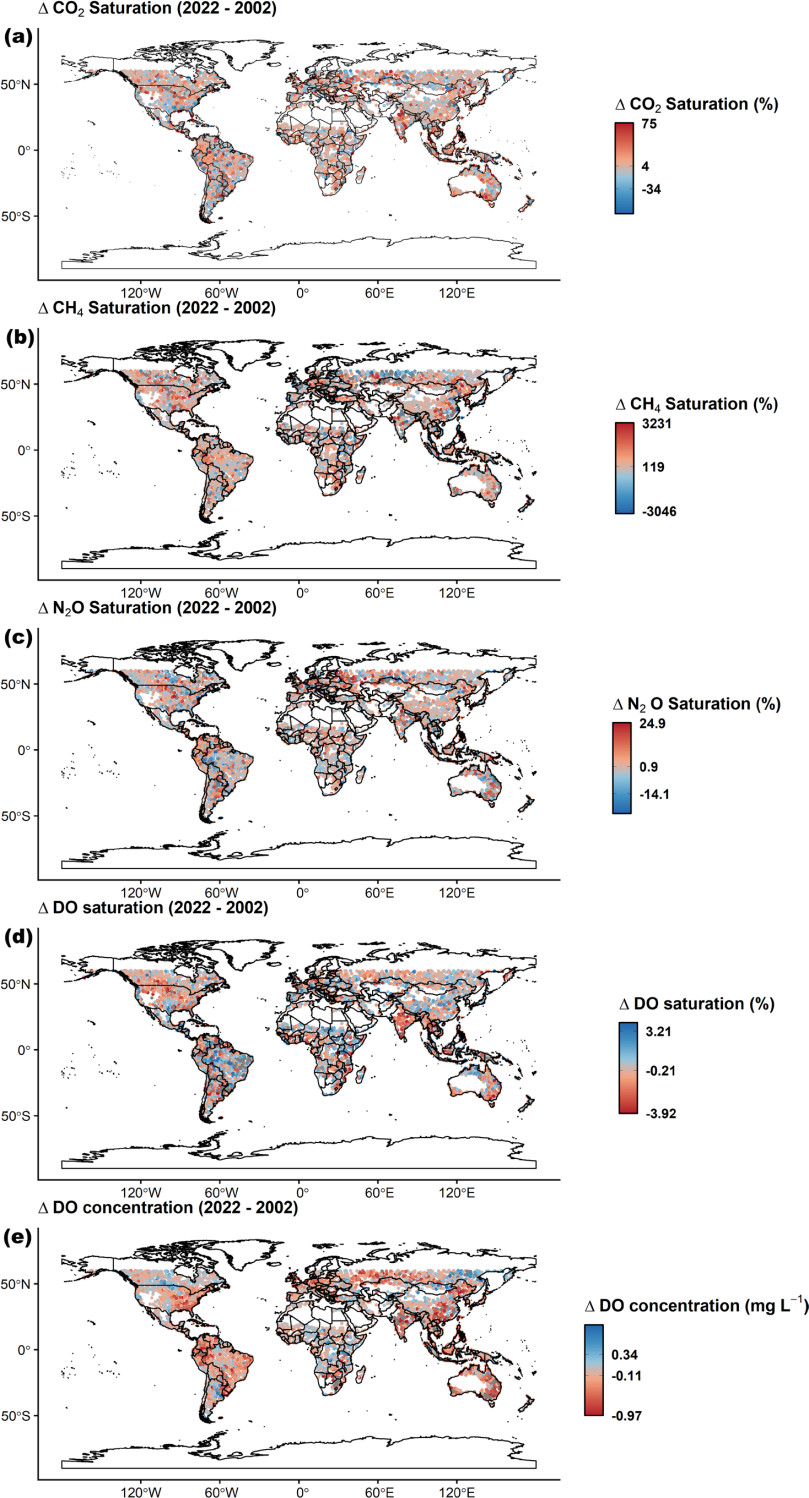
Global difference in annual means of CO_2_, CH_4_, N_2_O, and DO between 2002 and 2022. Map lines delineate study areas and do not necessarily depict accepted national boundaries. The red color gradient indicates positive differences, while the blue color gradient indicates negative differences. The colors are shifted in the DO case. Breaks on the legend represent the 5th, median, and 95th percentiles within the difference data.

Despite the limited number of measuring studies available for direct comparison, the observed regional temporal changes in some modeled riverine parameters align closely with those reported from regional long‐term field measurements. For instance, the modeled significant decadal increases in riverine water temperature (0.14°C–0.16°C dec^−1^) and decreases in DO concentrations (0.04–0.058 mg L^−1^ dec^−1^) for North America and Europe (Table 1) fall within the median decadal ranges (0.16°C–0.27°C and 0.034–0.059 mg L^−1^) reported for 796 rivers across the United States and Central Europe from 1981 to 2019 (Zhi et al. 2023). Additionally, our results show that the magnitude of temporal changes in riverine GHG supersaturation and deoxygenation exhibited distinct catchment scale patterns of either increasing or decreasing trends (Figure 4). Similar to our findings, several field‐based studies have shown contrasting catchment scale trends in either GHGs or DO (Jones et al. 2003; Ran et al. 2021). For instance, an analysis of 417 rivers across the contiguous United States over a 22‐year period (1973–1994) revealed a net CO_2_ decline, and net DO increases, but also significant catchment scale variation, with contrasting trends at some sites (Jones et al. 2003). Likewise, CO_2_ emissions from Chinese rivers showed a declining trend between the 1980s and 2010s, yet rivers within the Tibetan Plateau region exhibited increased emissions due to glacier melt and expanded stream networks (Ran et al. 2021). These examples highlight that the net regional and global trend, whether decline or increase, may often mask substantial heterogeneity at catchment scales. Still, the global median temporal changes over the 20 years indicated a general increase in fluvial GHG supersaturation (Figure 4a–c) and a decrease in DO (Figure 4d,e).

### 3.3 Combined Land Use and River Warming Effects on Modeled Riverine GHG Supersaturation and Deoxygenation

**FIGURE 5:**
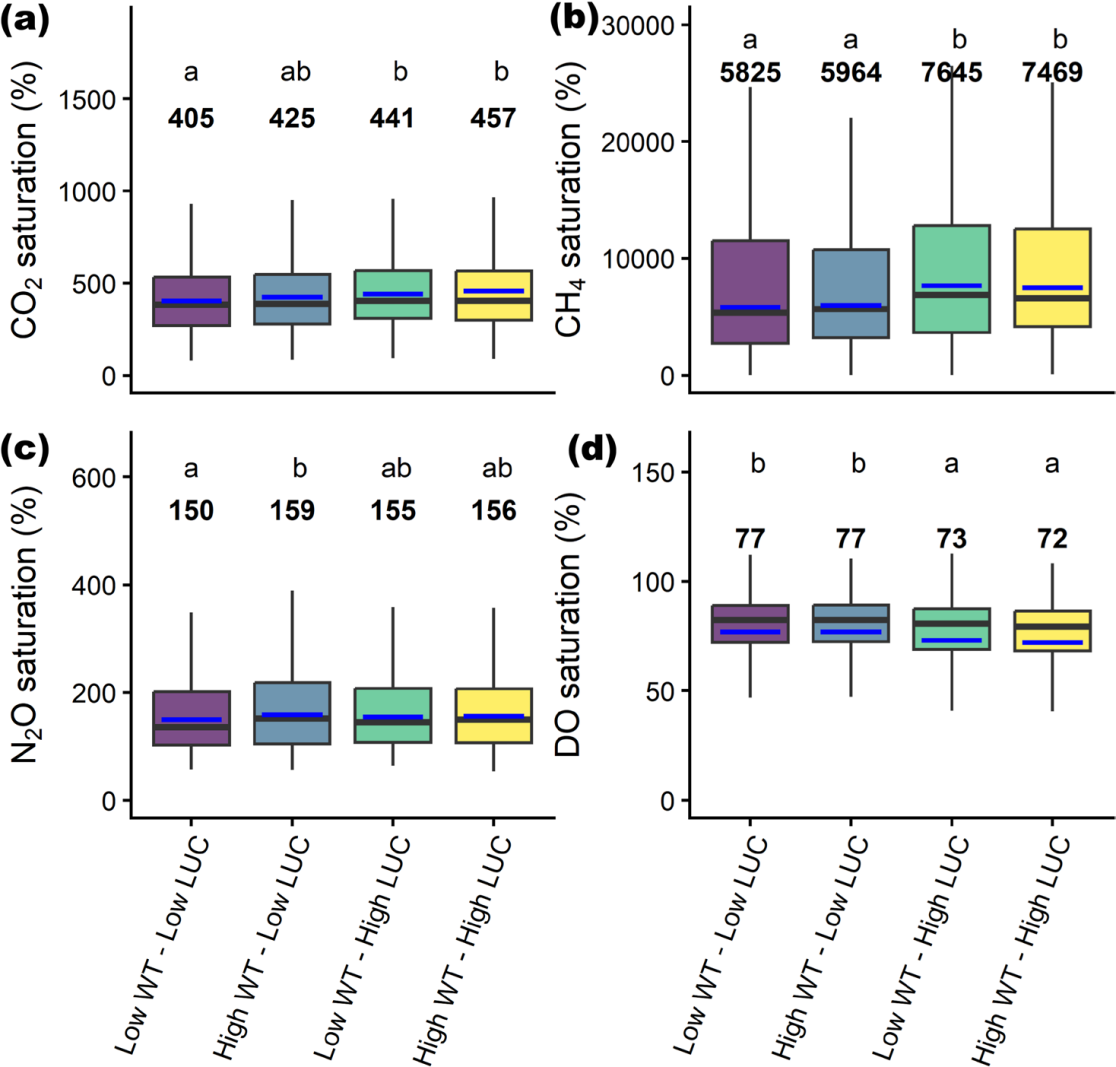
Comparison of modeled GHG and DO saturation across the 5084 catchments grouped by different levels of water temperature (WT) and anthropogenic land use changes (LUC) from 2002 to 2019. Blue lines and text represent the group means, and the letters above the boxplots indicate significant differences in mean values, as determined by Tukey's post hoc test from linear mixed‐effects models.

**FIGURE 6:**
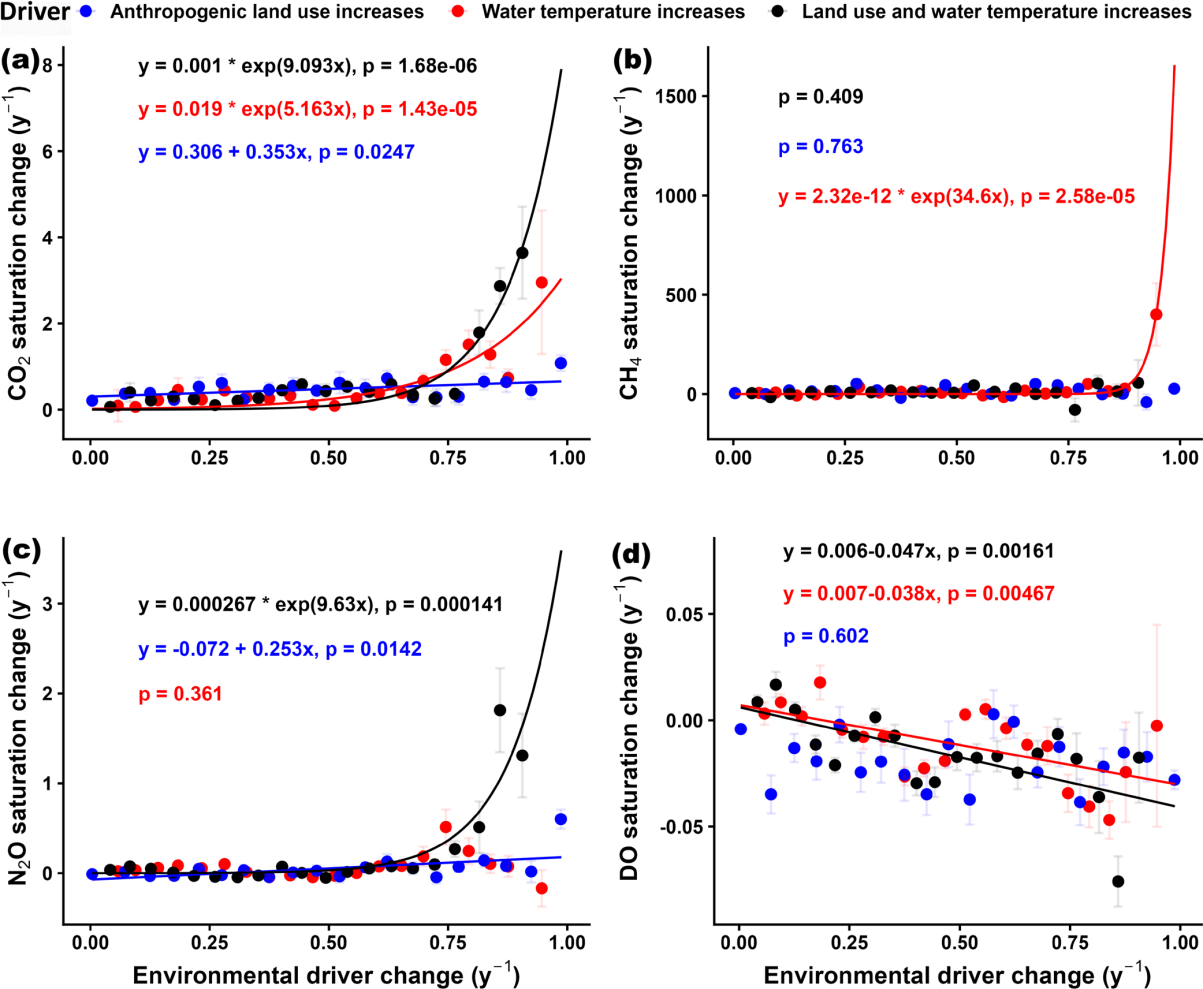
Bivariate relationships between annual rates of CO_2_, CH_4_, N_2_O, and DO saturation across 5084 catchments and concurrent changes in water temperature (red; scaled 0–1), anthropogenic land use (blue; scaled 0–1), and their combined effect (black; scaled 0–1). Data were binned into 20 equal‐width intervals (mean ± SE) to highlight underlying trends. Equations indicate significant (*p* < 0.05) relationships that follow exponential or linear forms.

Consistent with regional and global scale patterns, river warming and anthropogenic land use expansion showed mainly positive relationships with increased riverine GHG supersaturation and deoxygenation at the catchment scale (Figures 5 and 6). These environmental pressures predominantly exhibited synergistic rather than antagonistic effects (Figures 5 and 6), suggesting that warming and land use changes interact to amplify their impacts on fluvial ecosystems even at smaller catchment scales. For instance, streams experiencing greater increases in both river warming and upstream anthropogenic land use expansion had, on average, 1644% higher CH_4_ supersaturation, 52% higher CO_2_ supersaturation, and 5% greater DO undersaturation compared to those with lower changes in these environmental pressures (Figure 5a,b,d). In contrast to the other GHGs, instream N_2_O saturation showed only minor differences across these groups, though levels remained lower in catchments with limited anthropogenic expansion and river warming (Figure 5c). Streams experiencing higher rates of both warming and anthropogenic land use expansion were primarily concentrated in mid‐ to high‐latitude regions of the Northern Hemisphere and lower‐latitude regions of the Southern Hemisphere. Catchments with minimal increases in water temperature and anthropogenic land use expansion were predominantly located in high‐latitude Northern Hemisphere regions and equatorial tropical zones (Figure S9).

Further bivariate analyses examining how catchment‐scale temporal changes in GHGs and DO relate to shifts in environmental pressures also revealed consistently positive links (Figure 6). Overall, river warming exerted stronger positive effects on GHG supersaturation and deoxygenation than anthropogenic land use change (Figure 6a,b,d). Based on this finding, we hypothesize that climatic warming may be progressing more rapidly than land use shifts, thereby resulting in stronger effects on fluvial GHG supersaturation and deoxygenation rates. Among the GHGs, CH_4_ supersaturation was most sensitive to these thermal changes, showing pronounced exponential increases at sites with higher rates of river warming (Figure 6b). Such increases in riverine CH_4_ supersaturation with warming have also been reported in other freshwater ecosystems (Y. Zhu et al. 2020). Mechanistically, the stronger positive relationship between CH_4_ supersaturation and water temperature, relative to the other two GHGs, may indicate that CH_4_ production through methanogenesis increases more strongly with rising water temperature than do CO_2_ and N_2_O production processes such as respiration, nitrification, and denitrification. This conclusion aligns with other studies that have shown methanogenesis generally exhibits higher *Q*_10_ values (1.2–16) compared to denitrification (2.3–3.6) and respiration (1.5–2.3), indicating its greater temperature sensitivity, though these values can vary widely across ecosystems and conditions (Bååth 2018; Elsgaard et al. 2016; Waldo et al. 2021).

Still, catchments experiencing both warming and increasing anthropogenic land use exerted the steepest exponential increases in fluvial CO_2_ and N_2_O supersaturation, along with linear increases in deoxygenation (Figure 6a,c,d). Past studies have shown that urban and agricultural areas tend to warm more rapidly than natural landscapes due to thermal pollution from wastewater, the heat‐retention capacity of built infrastructure, and reduced riparian canopy cover (Liu, Zhan, et al. 2022; Marcotullio et al. 2021; Meng et al. 2023). These mechanisms likely explain the co‐occurrence of river warming and anthropogenic land use expansion observed in this study, which mainly exerted synergistic effects on riverine GHG supersaturation and deoxygenation (Figures 5 and 6).

Moreover, elevated nutrient and labile organic carbon inputs in streams within urban‐ and cropland‐dominated catchments are known to enhance biogenic GHG production and oxygen consumption processes (Piatka et al. 2021; Upadhyay et al. 2023; W. Xu et al. 2024). These substrate enrichments in surface waters may also be elevated under warmer conditions, which have been shown to accelerate carbon and nutrient release from stream sediments (Duan and Kaushal 2013; Zhou et al. 2016). Such mechanisms may explain the observed trends in instream DIN (NH_4_ and NO_3_), TN, and DOC concentrations, which were up to 1.6 times higher in streams experiencing increased river warming and anthropogenic land expansion than those experiencing the lowest changes in warming and land use expansion (Figure S10). Concurrently, lower DOC:TN ratios, often indicative of anthropogenic alteration of riverine stoichiometry (e.g., Wachholz et al. 2023), were also prevalent at these sites (Figure S10). Such declines in fluvial C:N ratios are known to stimulate microbial GHG production and DO consumption (Helton et al. 2015; Taylor and Townsend 2010), providing a mechanistic link between stoichiometric shifts and the observed increases in GHG supersaturation and deoxygenation.

### 3.4 Roles of Direct and Indirect Drivers on Temporal Trends in GHG Supersaturation

**FIGURE 7:**
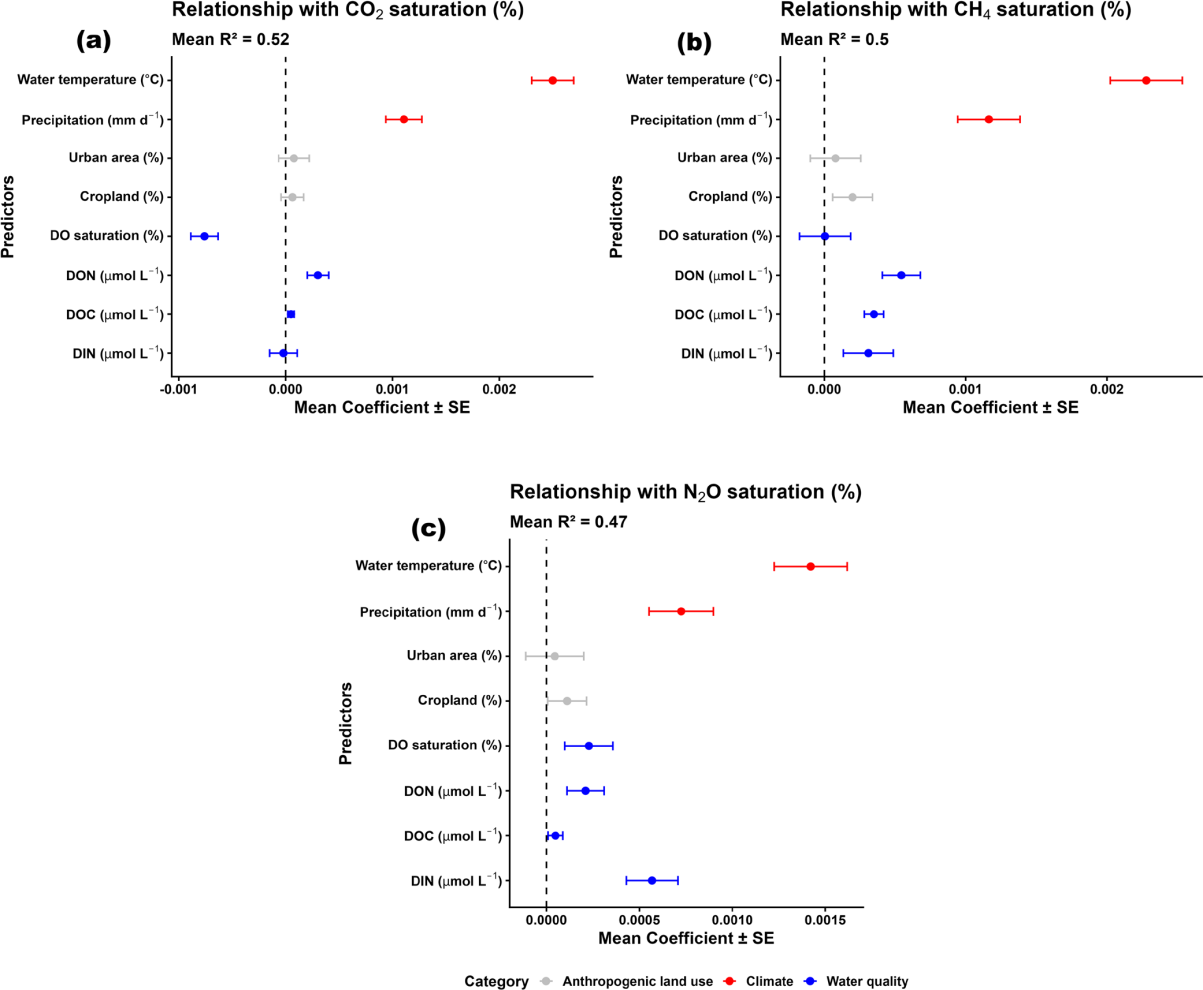
Multivariate analyses using elastic net regression models showing significant relationships between the direct (carbon and nitrogen substrates) and indirect drivers (water temperature, precipitation, and land use) of temporal changes in GHG supersaturation from the modeled dataset. Standardized coefficients for each predictor represent mean ± SE values across all the 5084 catchments, with the different colors indicating the predictor type that is, red (climatic), grey (land use) and blue (water quality). *R* ^2^ indicates average performance across all the 5084 catchments.

The multivariate analyses from the elastic net regression models at the catchment scale revealed clear differences in the relative importance of indirect versus direct drivers of GHG supersaturation (overall model performance: *r* ^2^ = 0.47–0.52). Climatic predictors emerged as the most important factors driving temporal increases in global riverine GHG supersaturation, relative to land use and water quality parameters (Figure 7). Among these, rising water temperatures exhibited the most pronounced positive relationship with increasing GHG supersaturation, while increasing precipitation showed a similarly positive relationship. These relationships are consistent with established mechanisms: elevated temperatures accelerate microbial activity that drives fluvial GHG production (e.g., Borges et al. 2018; Mwanake et al. 2023), and precipitation events deliver pulses of dissolved GHGs from surrounding terrestrial landscapes into rivers, amplifying supersaturation levels (e.g., Piatka et al. 2024).

Changes in substrate availability and DO were similarly important in explaining temporal changes in fluvial GHG supersaturation. Such relationships suggest that increased substrate availabilities and DO dynamics may favor instream processes responsible for GHG supersaturation, such as respiration, methanogenesis, and nitrification–denitrification (e.g., Battin et al. 2023; Mwanake et al. 2026; Stanley et al. 2016). For example, increases in both CO_2_ and CH_4_ supersaturation were positively associated with DOC (Figure 7a,b), and its more labile fraction, DON, while N_2_O showed the strongest positive relationship with DIN, while additionally being positively linked to DON (Figure 7c).

Riverine DO exhibited contrasting relationships with the three GHGs (Figure 7). Specifically, DO was on average negatively related to CO_2_, positively linked to N_2_O, and showed variable effects on CH_4_ that were comparatively minor (mean coefficient close to zero). These patterns reflect the different biogeochemical pathways linking fluvial DO dynamics to GHGs, which may explain our overall findings. On the one hand, oxygen depletion has been shown to enhance microbial respiration and CO_2_ release (e.g., Borges et al. 2018), while oxygen availability supports nitrification and N_2_O production (e.g., Mwanake, Gettel, et al. 2025). On the other hand, fluvial CH_4_ dynamics depend on both anoxic methanogenesis and oxic methane oxidation (Stanley et al. 2016), which may explain the site‐specific variability we observed. Yet, as CO_2_ is always the largest contributor to fluvial CO_2_ equivalent emissions (Lauerwald et al. 2023), its positive links with declining DO conditions suggest that these two adverse environmental outcomes may tend to co‐occur.

Cropland and urban expansion also exhibited positive relationships with all three GHGs, yet their effects were consistently weaker than those of climatic and water‐quality changes (Figure 7). We argue that rapid shifts in climate and water‐quality parameters, relative to the slower pace of land use transitions may explain their stronger influence on GHG supersaturation across the 20‐year period, in line with our earlier hypothesis. However, apart from their individual influences, it is highly likely that the effects of these drivers co‐vary. For instance, extreme precipitation events have been shown to favor terrestrial substrate inflows to streams (Mwanake et al. 2022; Piatka et al. 2024), while anthropogenic land uses also contribute to substrate enrichment in stream ecosystems, further intensifying substrate‐driven GHG emissions or oxygen depletion (Borges et al. 2018; Mwanake et al. 2019, 2022, 2023; Piatka et al. 2021; W. Xu et al. 2024).

### 3.5 Global Implications of Combined Warming and Anthropogenic Land Use Expansion on GHG Supersaturation

Our analysis of the modeled river dataset, derived from remote‐sensing observations from 2002 to 2022, shows that global riverine GHG supersaturation and deoxygenation are increasing. This is potentially driven by the synergistic effects of warming rivers, increased precipitation, and the expansion of cropland and urban areas that favor elevated instream biogenic processes. Given that global land use change projections under various climate scenarios indicate a substantial expansion of fertilizer‐intensive croplands and urban areas by the end of the 21st century (Alexander et al. 2018; X. Li et al. 2019), the increasing rates of riverine GHG supersaturation and deoxygenation shown in this study are likely to persist in the future.

That said, the simultaneous alteration of riverine GHG supersaturation and deoxygenation by human‐induced global warming and expansion of anthropogenic land use highlights the potential of human interventions to enable concrete measures for mitigating these two critical environmental problems. Such measures may involve expanding or preserving forested areas, which could help reduce riverine GHG supersaturation and deoxygenation. The importance of land use conversion as a potential mitigation measure was also demonstrated in a recent study quantifying Chinese inland waters GHG emissions (Ran et al. 2021). In that study, Ran et al. (2021) found that total inland water CO_2_ emissions declined markedly, from 138 ± 31 Tg C year^−1^ in the 1980s to 98 ± 19 Tg C year^−1^ in the 2010s, representing a ~29% reduction. They attributed this decrease partly to the widespread reforestation programs in China, which reduced riverine CO_2_ supersaturation and efflux.

Using the GHG emission rates from 2002 as a starting point and those of 2022 as the end point, and assuming no interannual variability, we estimate that maintaining the 2002 rates over the 20‐year period could have prevented the release of approximately 1.5 Pg CO_2_‐equivalent (or 0.075 Pg CO_2_‐eq per year), primarily emitted as CO_2_ (96%). We argue that this amount may reflect the anthropogenic share of riverine GHG emissions over the two decades, primarily driven by land use and climate‐change impacts. We also contend that this value is likely a conservative estimate, as we did not account for increases in riverine surface areas and gas transfer velocities due to the yearly increase in extreme precipitation events anticipated with global climate change (Thackeray et al. 2022).

### 3.6 Limitations of Our Modeling Approach

While our global reconstruction of instream water temperature, GHGs, DO, and other water quality parameters revealed meaningful trends consistent with expected changes in riverine biogeochemistry under anthropogenic pressures, the absolute values are subject to several sources of uncertainty that we could not fully address. As a result, some uncertainties may remain embedded in the final predictions. These uncertainties primarily arise from the limited availability of globally distributed, continuous datasets for robust model training and validation, as well as several modeling assumptions we made. Specifically, sub‐daily fluvial GHG data and corresponding remote sensing products remain scarce globally, which constrained the training of our models to a daily resolution. As a result, finer‐scale fluvial GHG and DO dynamics, such as diel variability or the effects of short‐term hydrological events, were not represented, which have been shown to have a significant effect on overall fluxes (Gómez‐Gener et al. 2021; Piatka et al. 2024).

The assumption that daily GHG and water‐quality parameters can be predicted from the same‐day predictors derived from remote sensing may not always be feasible. Response times are likely to vary depending on site‐specific conditions, and processes such as land‐to‐river carbon and nutrient inputs often involve delays due to turnover in vegetation biomass, soils, and hydrological transport pathways. This limitation may partly explain the relatively low temporal skill observed for some parameters (Figure S4). Nevertheless, because we characterized broader annual trends that average out intra‐annual short‐term dynamics, we expect that potential biases in these dynamics only had a minimal impact on the overall outcomes of this study.

Another limitation of this study is the spatial scope of our modeling. In computing global annual means, we included only our modeled 5084 catchments, excluding other potential hotspots and coldspots of GHG and DO saturation. River catchments within deserts and parts of the Arctic were omitted, and while their influence may be relatively small at the global scale due to conditions that limit fluvial GHG emissions (e.g., very cold temperatures or little to no flowing water), their exclusion nonetheless reduced our ability to capture the full diversity of riverine environments. Other potential uncertainties include cloud removal in MODIS satellite products, which may not always yield expected results, leaving residual cloud artifacts that may have influenced predictions of riverine GHG and water quality trends.

That said, by integrating diverse global datasets with spatially and temporally explicit remote‐sensing products and applying machine‐learning models to capture complex relationships, our study represents an important first step toward quantifying long‐term global river responses to land use and climate change, thereby laying the foundation for future refinement and validation. Such improvements will involve the development of long‐term global distributed observatories dedicated to riverine GHG emissions, as recommended in several other studies (Battin et al. 2023; Lauerwald et al. 2023).

## Author Contributions

**Ralf Kiese:** writing – review and editing, conceptualization, investigation, resources. **Ricky Mwangada Mwanake:** conceptualization, writing – original draft, writing – review and editing, methodology, validation, visualization, formal analysis, data curation, investigation. **Elizabeth Gachibu Wangari:** writing – review and editing, methodology, data curation.

## Conflicts of Interest

The authors declare no conflicts of interest.

## Supporting information


**Data S1:** gcb70828‐sup‐0001‐TableS1‐S3‐FigureS1‐S10.pdf.

## Data Availability

The data that support the findings of this study are openly available in Zenodo at https://doi.org/10.5281/zenodo.18266557.
