# Supplementary material for: Rising Global Riverine Deoxygenation Rates and GHG Emissions Driven by the Synergistic Effects of Warming and Anthropogenic Land Use Expansion
Source: Glob Chang Biol. 2026 Mar 27;32(3):e70828. doi: 10.1111/gcb.70828 (PMC13022804; doi:10.1111/gcb.70828)
Supplement: Supplementary file 1 — Data S1: gcb70828‐sup‐0001‐TableS1‐S3‐FigureS1‐S10.pdf. [file GCB-32-e70828-s001.pdf]

Supplementary Information:

Title: Rising global riverine deoxygenation rates and GHG emissions driven by the synergistic effects of warming and anthropogenic land use expansion

Authors: *Ricky Mwangada Mwanake[1], Elizabeth Gachibu Wangari[1], Ralf Kiese [1]

[1]Karlsruhe Institute of Technology, Institute for Meteorology and Climate Research, Atmospheric Environmental Research (IMK-IFU), Kreuzeckbahnstrasse 19, Garmisch-Partenkirchen 82467, Germany

*Corresponding author email: ricky.mwanake2@kit.edu

Table S1: Summary of the 14 remotely sensed datasets used to predict spatial-temporal trends in GHG and water quality using the random forest algorithm. Also included are the inferred mechanistic links due to similar links with terrestrial soil parameters (dos Santos et al., 2025; Farella et al., 2022; Imtiaz et al., 2024; Kunkel et al., 2022).

Remotely sensed dataset	Bands used	Temporal coverage	Spatial Resolution (m)	Inferred mechanistic links with in-stream GHG and water quality dynamics	Data source
Daily Photosynthetically Active Radiation (3-Hourly)	GMT_0600_PAR	2000-02-24 –2024-10-24	500	Controls light availability that drives photosythetic and respiratory cycles within streams	https://developers.google.com/earth-engine/datasets/catalog/MODIS_062_MCD18C2#bands
	GMT_0900_PAR	2000-02-24 –2024-10-24			
	GMT_1200_PAR	2000-02-24 –2024-10-24			
	GMT_1500_PAR	2000-02-24 –2024-10-24			
	GMT_1800_PAR	2000-02-24 –2024-10-24			
	GMT_2100_PAR	2000-02-24 –2024-10-24			
Daily Enhanced Vegetation Index (EVI)	EVI	2000-02-24 –2023-02-17	463.313	Proxy for instream nutrient and organic matter	https://developers.google.com/earth-engine/datasets/catalog/MODIS_MOD09GA_006_EVI#bands
Daily Normalized Difference Vegetation Index (NDVI)	NDVI	2000-02-24 –2023-02-17	463.313	Proxy for instream nutrient and organic matter	https://developers.google.com/earth-engine/datasets/catalog/MODIS_MOD09GA_006_NDVI#bands
Daily Normalized Difference Water Index (NDWI)	NDWI	2000-02-24 –2023-02-17	463.313	Proxy for terrestrial-stream water hydrological connectivity	https://developers.google.com/earth-engine/datasets/catalog/MODIS_MOD09GA_006_NDWI
Daily Normalized Difference Snow Index (NDSI)	NDSI	2000-02-24 –2023-02-17	463.313	Proxy for terrestrial-stream water hydrological connectivity	https://developers.google.com/earth-engine/datasets/catalog/MODIS_MOD09GA_006_NDSI#bands
Daily Burn Area Index (BAI)	BAI	2002-07-04–2023-02-25	463.313	Proxy for instream nutrient and organic matter	https://developers.google.com/earth-engine/datasets/catalog/MODIS_MYD09GA_006_BAI#bands
SRTM-derived topographic diversity (D)	Constant		270	Proxy for terrestrial-stream water hydrological connectivity	https://developers.google.com/earth-engine/datasets/catalog/CSP_ERGo_1_0_Global_SRTM_topoDiversity#description
Elevation	Elevation		90	Proxy for terrestrial-stream water hydrological connectivity	https://developers.google.com/earth-engine/datasets/catalog/CGIAR_SRTM90_V4#bands
Stream order			Reach scale	Proxy for terrestrial-stream water hydrological connectivity	

Table S2: Summary of the measured global data on water temperature, water quality parameters, and GHG saturation used for model training and validation collected between 2000 and 2022. Also included is a summary of the predicted values from the random forest models for the specific site and date, compared with the measured data, along with the coefficient of determination (r^2) and the uncertainty in the predicted values, expressed as a percentage of the MAE relative to the measured mean. The total number of prediction values (4831) represents the maximum number of site-sampling date pairs in the measured values.

	**Measured values**			**Predicted values**				
**Variable**	**N**	**Range**	**Mean±SE**	**N**	**Range**	**Mean±SE**	r^2	**Uncertanity % (MAE/measured mean)**
Water temperature (°C)	4728	-2.07 - 37.5	14.87 ± 0.11	4831	1.31 - 34.43	15.57 ± 0.1	0.78	18.3
Conductivity (µS cm^{-1})	2536	2.7 - 2480	310.94 ± 6.67	4831	4.25 - 1623.68	291.85 ± 3.25	0.76	6.6
pH	3290	3.11 - 10.67	7.42 ± 0.01	4831	3.84 - 9.59	7.45 ± 0.01	0.62	16.5
DO (mg L^{-1})	2741	0.15 - 20.99	8.62 ± 0.05	4831	2.28 - 17.02	8.11 ± 0.03	0.59	14.0
DO saturation (%)	2578	0.27 - 211	75.38 ± 0.61	4831	2.78 - 155.31	75.27 ± 0.35	0.55	6.2
NH_4 (µmol L^{-1})	2478	0 - 3353	82.8 ± 3.81	4831	0 - 838.99	37.57 ± 1.1	0.75	15.6
NO_3 (µmol L^{-1})	2651	0.01 - 2520.89	114.77 ± 3.82	4831	0.03 - 942.52	67.89 ± 1.69	0.90	13.4
DOC (µmol L^{-1})	2882	8.33 - 7281.44	710.66 ± 15.99	4831	28.7 - 4380.21	493.52 ± 7.4	0.64	6.8
TN (µmol L^{-1})	913	0.01 - 2670.1	155.91 ± 7.58	4831	0.02 - 915.78	96.32 ± 1.33	0.70	13.5
TP (µmol L^{-1})	768	0.03 - 272.81	3.86 ± 0.57	4831	0.1 - 16.77	2.28 ± 0.02	0.53	47.2
CO_2 saturation (%)	3214	1.51 - 11979.88	641.76 ± 13.86	4831	32.34 - 4293.77	555.65 ± 6.82	0.59	7.2
CH_4 saturation (%)	4300	0 - 6672760.74	44177.43 ± 3746.4	4831	0 - 907078.03	20492.73 ± 652.53	0.50	9.1
N_2O saturation (%)	1983	0.95 - 20967.05	234.68 ± 13.14	4831	10.92 - 2547.95	204.03 ± 2.56	0.57	6.6

Table S3: Modeled annual means of water quality parameters, GHG saturations, and DO saturation from 5084 globally distributed catchments from 2002 to 2022. The catchments do not include desert or tundra regions (Indicated in Dinerstein et al., 2017).

	**Predicted annual reach scale values**					
**Variable**	**Number of reaches**	**Temporal duration**	**Range**	**Mean±SE**	r^2	**Uncertanity % (MAE/measured mean)**
Water temperature (°C)	5084	2002 - 2022	3.85 - 32.1	16.3 ± 0.01	0.78	18.3
Conductivity (µS cm^{-1})	5084	2002 - 2022	8.21 - 1100.5	220.81 ± 0.47	0.76	6.6
pH	5084	2002 - 2022	4.77 - 8.67	7.33 ± 0	0.62	16.5
DO (mg L^{-1})	5084	2002 - 2022	3.87 - 11.93	8.46 ± 0	0.59	14.0
DO saturation (%)	5084	2002 - 2022	11.9 - 112.62	77.93 ± 0.05	0.55	6.2
NH_4 (µmol L^{-1})	5084	2002 - 2022	0.19 - 334.01	12.44 ± 0.05	0.75	15.6
NO_3 (µmol L^{-1})	5084	2002 - 2022	0.12 - 624.37	29.3 ± 0.13	0.90	13.4
DOC (µmol L^{-1})	5084	2002 - 2022	44.62 - 2665.79	483.52 ± 0.98	0.64	6.8
TN (µmol L^{-1})	5084	2002 - 2022	0.72 - 525.58	83.36 ± 0.24	0.70	13.5
TP (µmol L^{-1})	5084	2002 - 2022	0.18 - 12.26	1.72 ± 0	0.53	47.2
CO_2 saturation (%)	5084	2002 - 2022	79 - 3181.91	535.57 ± 1.37	0.59	7.2
CH_4 saturation (%)	5084	2002 - 2022	0.28 - 197945.63	10467.35 ± 37.34	0.50	9.1
N_2O saturation (%)	5084	2002 - 2022	53.56 - 1044.52	174.61 ± 0.31	0.57	6.6

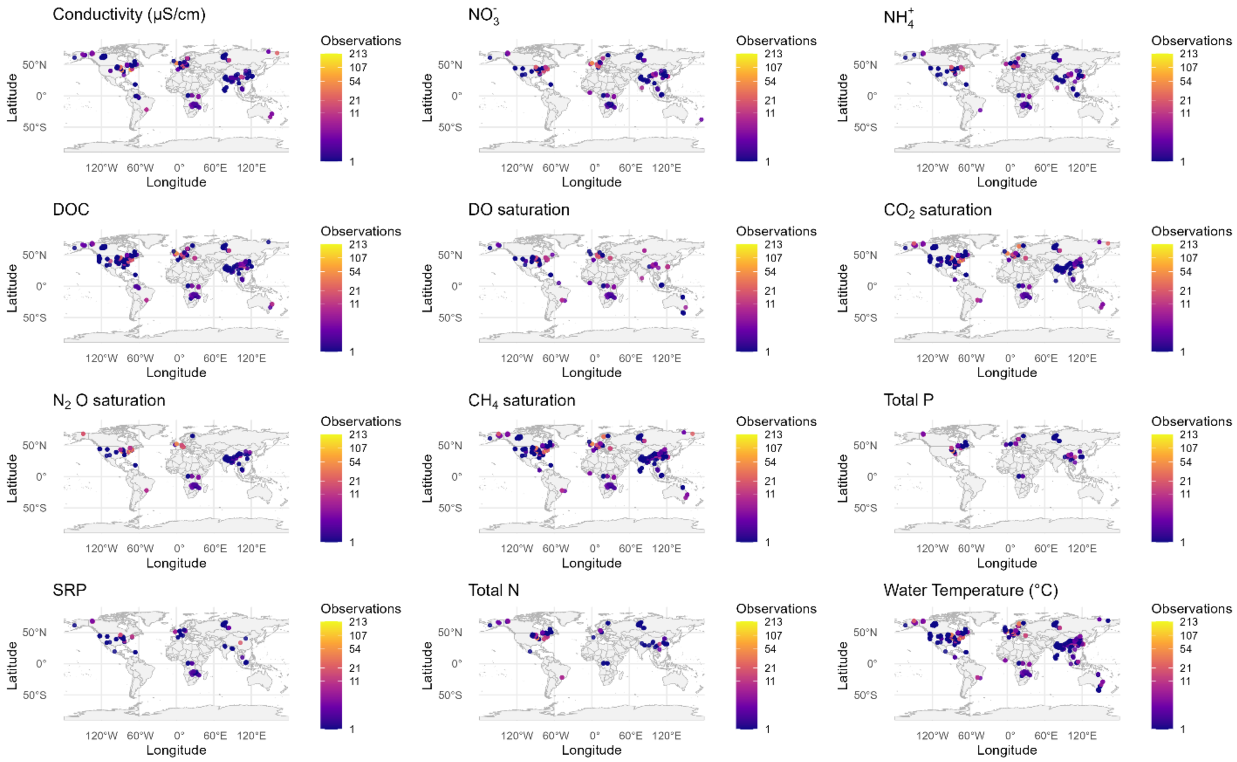

Figure S1: Spatial distribution of the 1085 catchments within the training and validation dataset. The color of the dots indicates the number of observations at each of the locations.

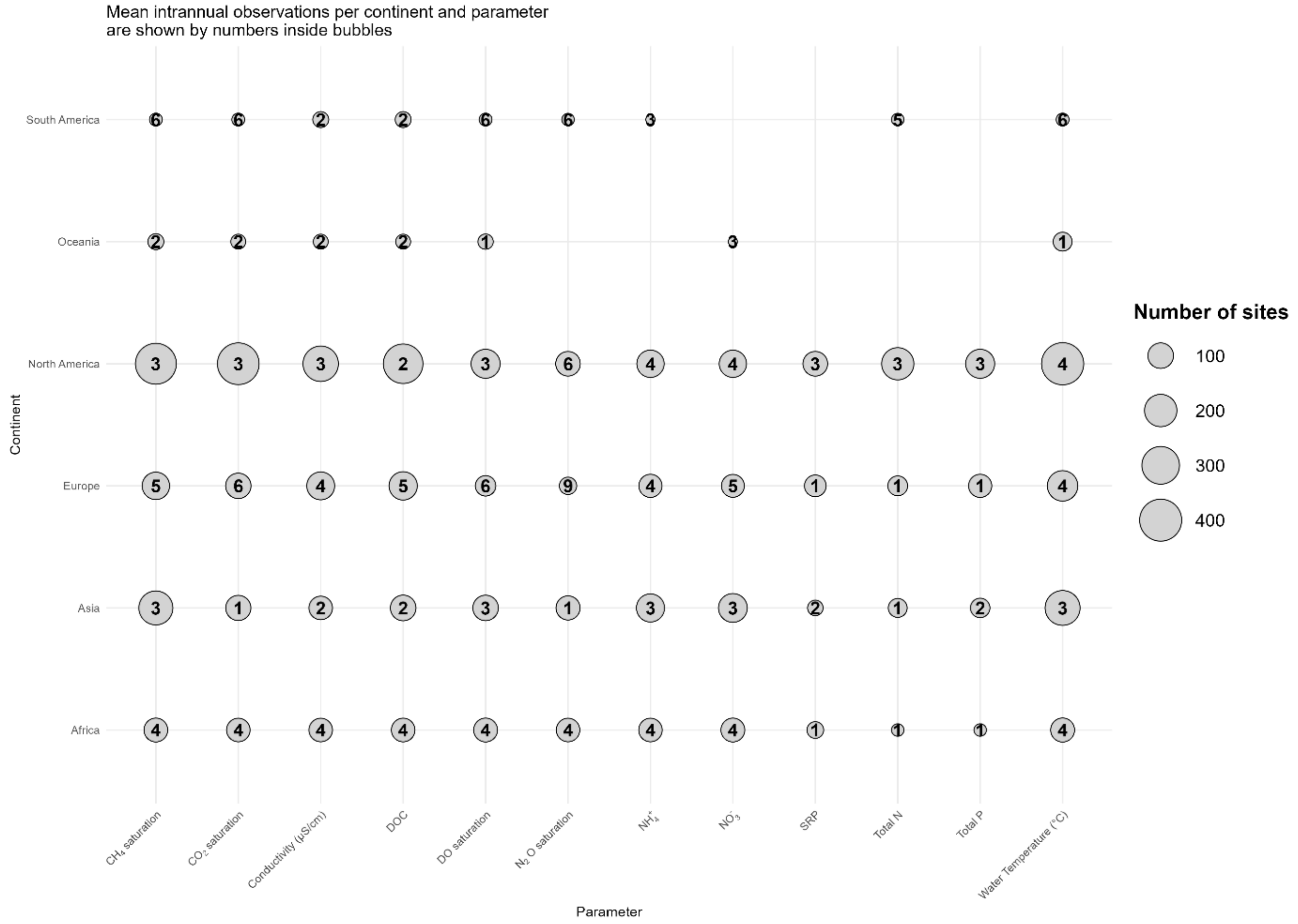

Figure S2: Continental distribution of the sites within the training and validation dataset. Numbers in the dots indicate the average number of intra-annual observations per parameter within each continent while the size of the dots indicates the total number of sites within each continent.

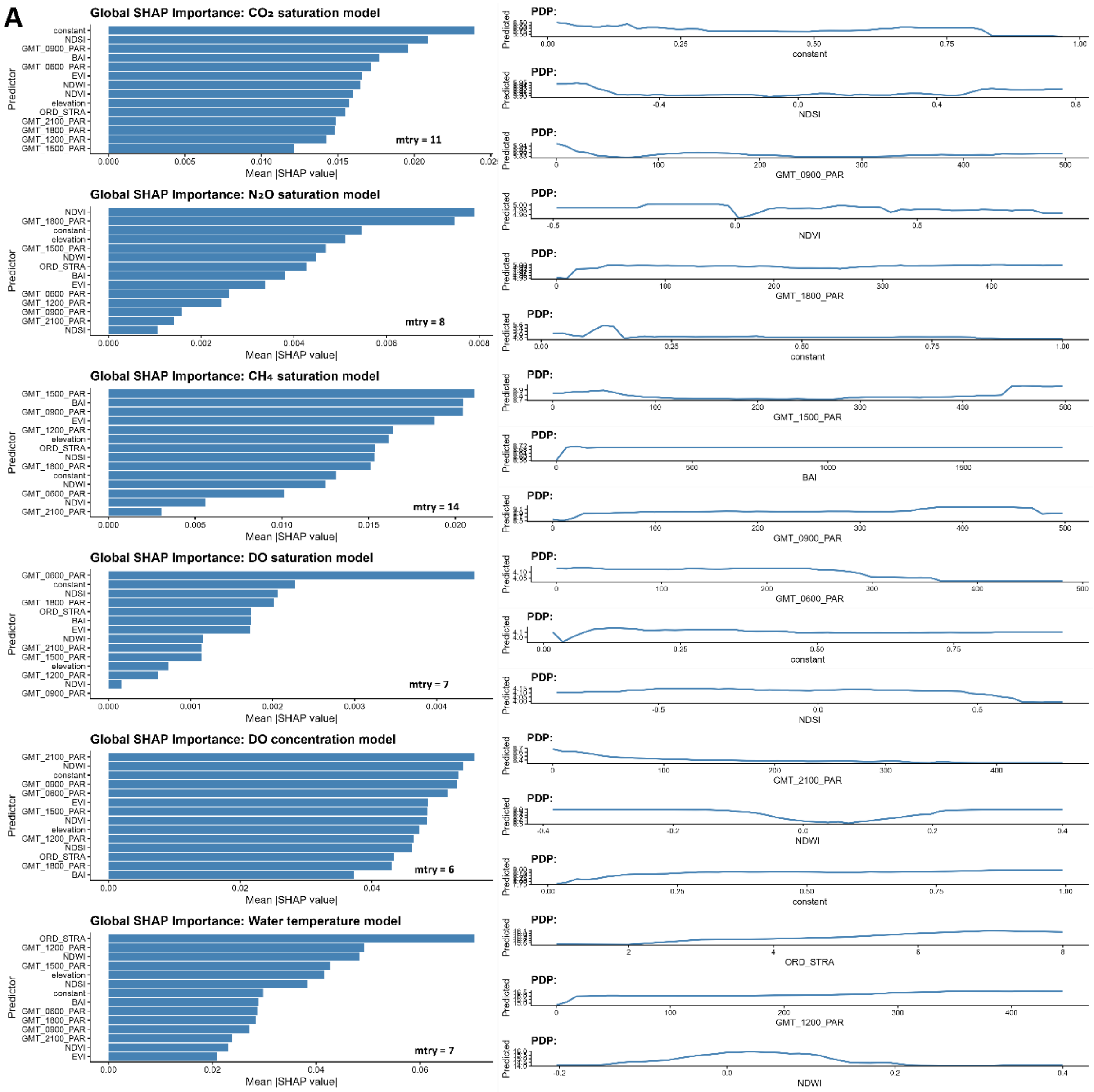
A
Global SHAP Importance: CO₂ saturation model
mtry = 11
Global SHAP Importance: N₂O saturation model
mtry = 8
Global SHAP Importance: CH₄ saturation model
mtry = 14
Global SHAP Importance: DO saturation model
mtry = 7
Global SHAP Importance: DO concentration model
mtry = 6
Global SHAP Importance: Water temperature model
mtry = 7
Predictor
Mean |SHAP value|
PDP:
Predicted
constant
NDSI
GMT_0900_PAR
NDVI
GMT_1800_PAR
GMT_1500_PAR
BAI
GMT_0600_PAR
GMT_2100_PAR
NDWI
ORD_STRA
GMT_1200_PAR

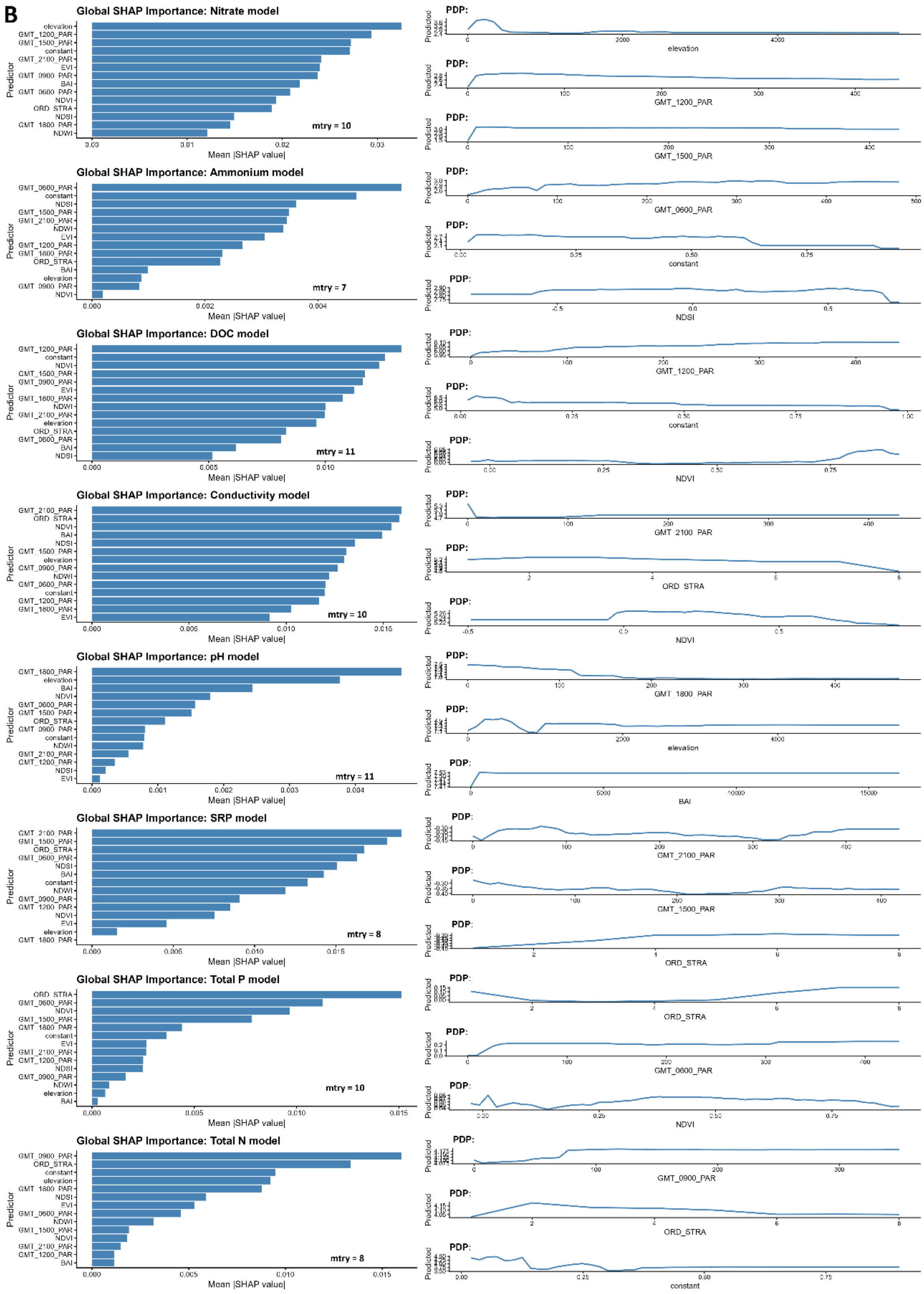

Figure S3A, B: Left Panel displays the relative importance of all 14 predictors from the Random Forest models predicting the **A**) GHGs, DO, water temperature and **B)** other water quality parameters, while Right Panel shows partial dependence plots for the three most influential predictors. The most important hyperparameter (mtry= number of predictors in each tree node) calibrated during the model construction is also included.

The observed relationships between the top three predictors and response variables mainly aligned well with mechanistic expectations, while others had both positive and negative relationships along the predictor ranges (Figure S3).

To mention a few examples, the topographic diversity constant, a potential proxy for biodiversity, was negatively associated with fluvial CO_2, N_2O supersaturation, TN, DO and NH_4 but positively associated with DO. This suggests that more biodiverse landscapes, potentially representing more natural landscapes, tend to have lower fluvial GHGs and nutrients and higher DO concentrations compared to less diverse regions that may be more human influenced.

Fluvial DO concentrations exhibited a negative relationship with photosynthetically active radiation (PAR), likely reflecting reduced solubility under warmer conditions due to increased radiation. In contrast, water temperature, CH_4, N_2O, TN and TP showed the opposite trend, increasing with PAR.

The negative relationship between stream order and TN agrees with established mechanisms, smaller streams often have higher TN due to stronger connectivity with groundwater and surrounding terrestrial ecosystems compared to larger rivers. In addition, stream order was also positively associated to water temperature, suggesting that larger rivers may warm faster due to their large surface area compared to smaller streams.

Some relationships with vegetation indices further supported mechanistic interpretations. For example, the positive association between NDVI and DOC suggests higher inputs of terrestrial OM supports higher DOC concentration.

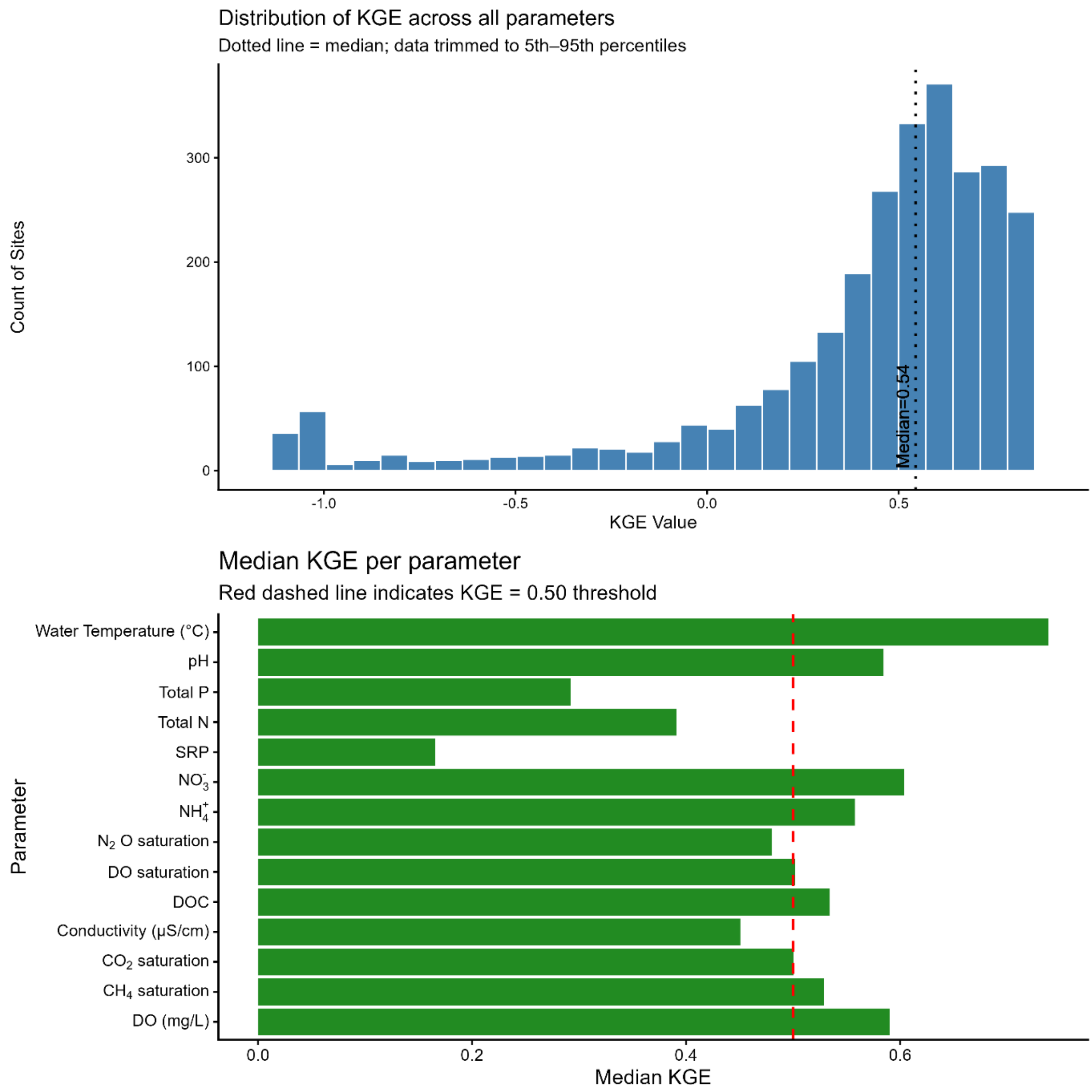

Figure S4: Distribution and parameter-wise performance of the temporal skill of the random Forest models evaluated using the Kling-Gupta Efficiency (KGE). The top panel shows histograms of KGE values across all sites and response variables, with the dotted line indicating the median. The bottom panel presents the median KGE for each response variable.

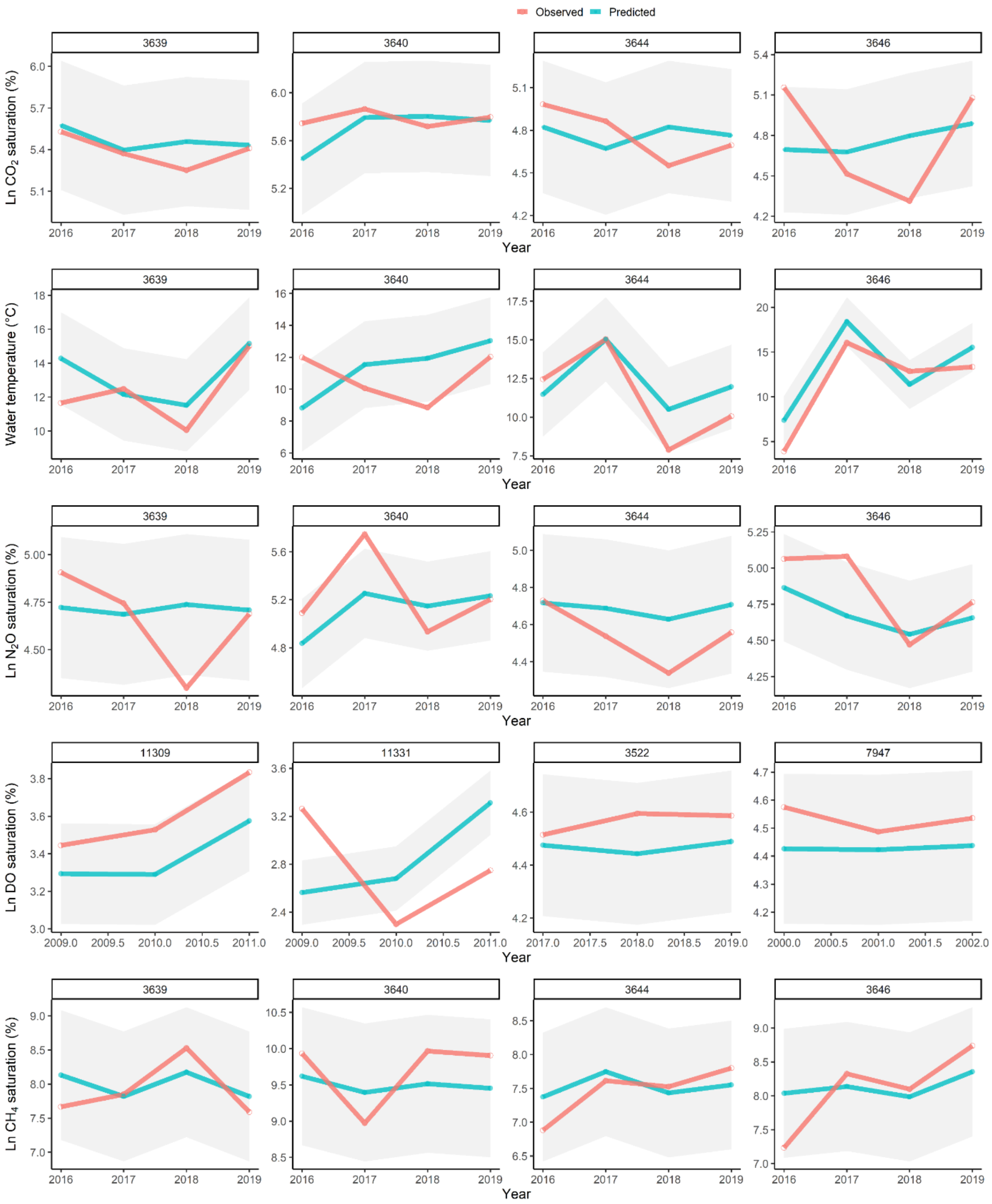

Figure S5: Comparison of actual and predicted annual means of ln CO_2, CH_4, N_2O, and DO % saturation, as well as water temperature, using the test dataset that was not utilized for model training at four sites with multi-year data. The test dataset had only these 4 sites with the multiyear data, which limited the comparison. The grey error bounds indicate the MAE of the predictions from the random forest models. The numbers at the top of the graphs refer to site codes from the GRiMeDB database (Stanley et al., 2023).

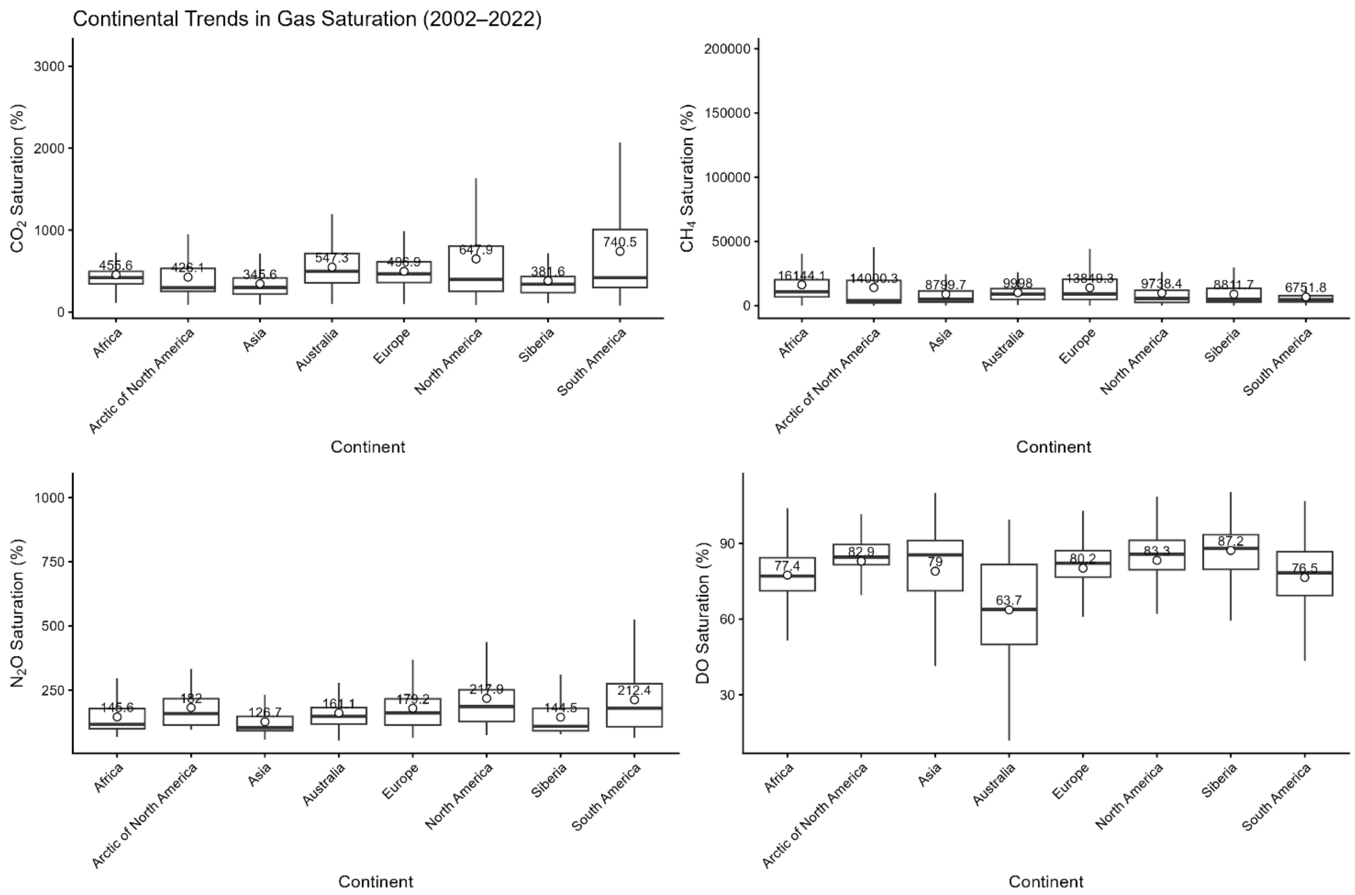

Figure S6: Continental summaries of the modeled GHG saturations from 2002 to 2022. Boxplots show the median, 25th and 75th percentiles; while dots and numbers indicate the mean value.

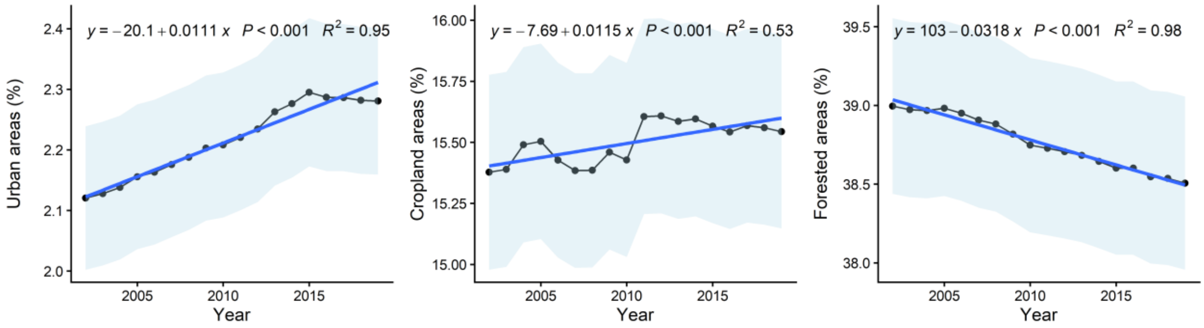

Figure S7: Global upstream land use trends of urban areas, croplands and forests percentage averaged across the 5084 modeled catchments for the period 2002 to 2019. The land use data was extracted from the HILDA+ dataset (Winkler et al., 2021).

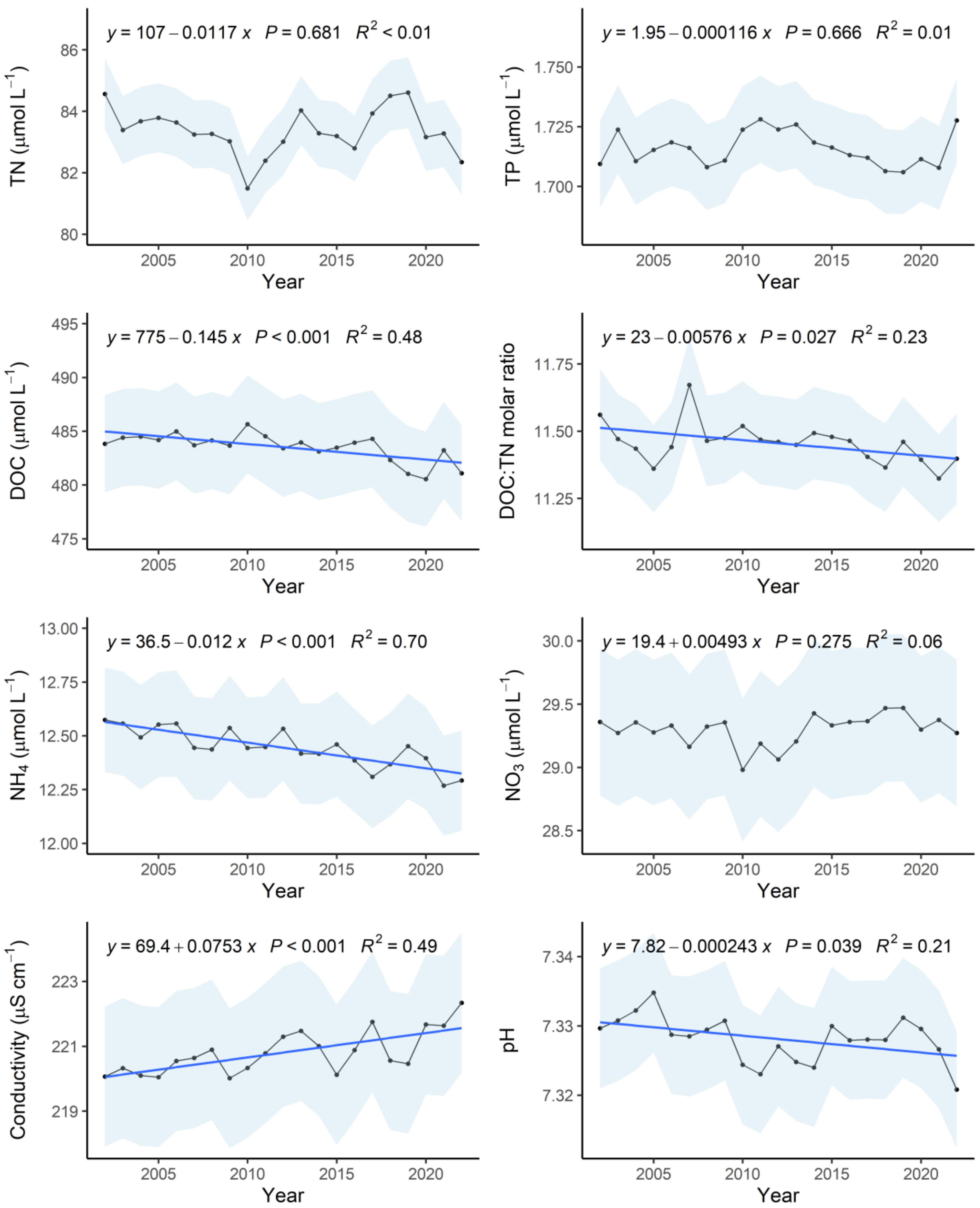

Figure S8: Modeled global annual mean ±SE trends of water quality parameters from the 5084 sites.

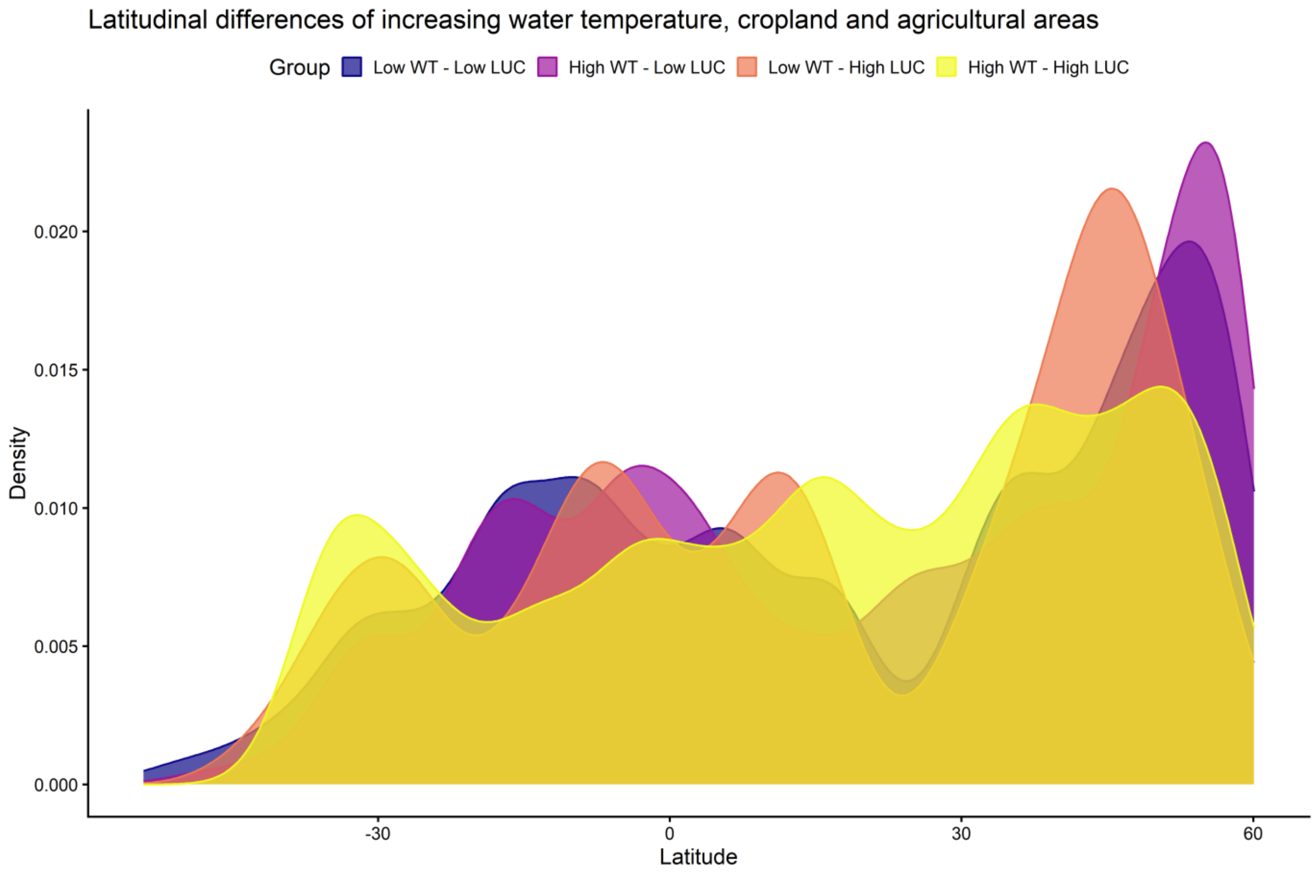

Figure S9: Density plots indicating the latitudinal distribution of the modeled sites with the different levels of river warming and anthropogenic land use changes.

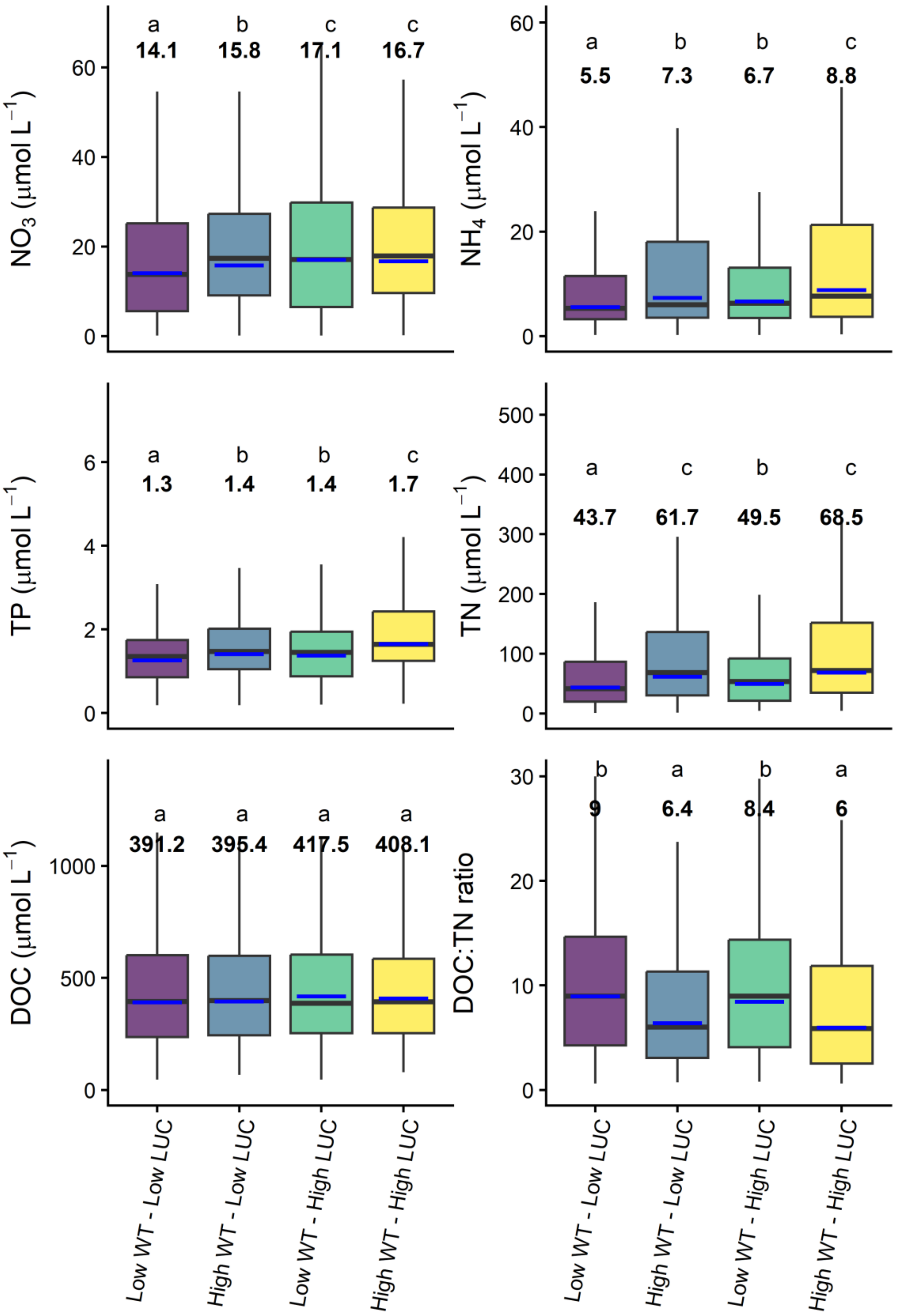

Figure S10: Comparison of water quality parameters across the 5084 catchments grouped by different levels of water temperature and anthropogenic land use changes from 2002 to 2022. Blue lines and text represent the group means, and the letters on top of the boxplots indicate significant differences in mean values, as determined by the Tukey post hoc test from linear mixed-effects models.

References

Dinerstein, E., Olson, D., Joshi, A., Vynne, C., Burgess, N. D., Wikramanayake, E., Hahn, N., Palminteri, S., Hedao, P., Noss, R., Hansen, M., Locke, H., Ellis, E. C., Jones, B., Barber, C. V., Hayes, R., Kormos, C., Martin, V., Crist, E., … Saleem, M. (2017). An Ecoregion-Based Approach to Protecting Half the Terrestrial Realm. *BioScience*, *67*(6), 534–545. https://doi.org/10.1093/biosci/bix014

dos Santos, E. P., Moreira, M. C., Fernandes-Filho, E. I., Demattê, J. A. M., Santos, U. J. dos, Moura-Bueno, J. M., Cruz, R. R. P., Silva, D. D. da, & Sampaio, E. V. de S. B. (2025). Integrating satellite radar vegetation indices and environmental descriptors with visible-infrared soil spectroscopy improved organic carbon prediction in soils of semi-arid Brazil. *Geoderma*, *457*, 117288. https://doi.org/10.1016/j.geoderma.2025.117288

Farella, M. M., Barnes, M. L., Breshears, D. D., Mitchell, J., van Leeuwen, W. J. D., & Gallery, R. E. (2022). Evaluation of vegetation indices and imaging spectroscopy to estimate foliar nitrogen across disparate biomes. *Ecosphere*, *13*(3). https://doi.org/10.1002/ecs2.3992

Imtiaz, F., Farooque, A. A., Randhawa, G. S., Wang, X., Esau, T. J., Acharya, B., & Hashemi Garmdareh, S. E. (2024). An inclusive approach to crop soil moisture estimation: Leveraging satellite thermal infrared bands and vegetation indices on Google Earth engine. *Agricultural Water Management*, *306*, 109172. https://doi.org/10.1016/j.agwat.2024.109172

Kunkel, V. R., Wells, T., & Hancock, G. R. (2022). Modelling soil organic carbon using vegetation indices across large catchments in eastern Australia. *Science of The Total Environment*, *817*, 152690. https://doi.org/10.1016/j.scitotenv.2021.152690

Winkler, K., Fuchs, R., Rounsevell, M., & Herold, M. (2021). Global land use changes are four times greater than previously estimated. *Nature Communications*, *12*(1). https://doi.org/10.1038/s41467-021-22702-2

Stanley, E. H., Loken, L. C., Casson, N. J., Oliver, S. K., Sponseller, R. A., Wallin, M. B., Zhang, L., & Rocher-Ros, G. (2023). GRiMeDB: the Global River Methane Database of concentrations and fluxes. *Earth System Science Data*, *15*(7), 2879–2926. https://doi.org/10.5194/essd-15-2879-2023
